# Glutathione-Responsive Tannic Acid-Assisted FRET Nanomedicine for Cancer Therapy

**DOI:** 10.3390/pharmaceutics15051326

**Published:** 2023-04-24

**Authors:** Partha Laskar, Anupam Dhasmana, Sudhir Kotnala, Meena Jaggi, Murali M. Yallapu, Subhash C. Chauhan

**Affiliations:** 1 Department of Immunology and Microbiology, School of Medicine, University of Texas Rio Grande Valley, McAllen, TX 78504, USA; 2 South Texas Center of Excellence in Cancer Research, School of Medicine, University of Texas Rio Grande Valley, McAllen, TX 78504, USA; 3 The Ångström Laboratory, Macromolecular Chemistry, Department of Chemistry, Uppsala University, 751 21 Uppsala, Sweden; 4 Cancer Research Institute, Himalayan School of Biosciences, Swami Rama Himalayan University, Dehradun 248016, India

**Keywords:** combination cancer therapy, camptothecin, curcumin, tannic acid, glutathione-responsive nanoparticle, FRET

## Abstract

In cancer combination therapy, a multimodal delivery vector is used to improve the bioavailability of multiple anti-cancer hydrophobic drugs. Further, targeted delivery of therapeutics along with simultaneous monitoring of the drug release at the tumor site without normal organ toxicity is an emerging and effective strategy for cancer treatment. However, the lack of a smart nano-delivery system limits the application of this therapeutic strategy. To overcome this issue, a PEGylated dual drug, conjugated amphiphilic polymer (CPT-S-S-PEG-CUR), has been successfully synthesized by conjugating two hydrophobic fluorescent anti-cancer drugs, curcumin (CUR) and camptothecin (CPT), through an ester and a redox-sensitive disulfide (-S-S-) linkage, respectively, with a PEG chain via in situ two-step reactions. CPT-S-S-PEG-CUR is spontaneously self-assembled in the presence of tannic acid (TA, a physical crosslinker) into anionic, comparatively smaller-sized (~100 nm), stable nano-assemblies in water in comparison to only polymer due to stronger H-bond formation between polymer and TA. Further, due to the spectral overlap between CPT and CUR and a stable, smaller nano-assembly formation by the pro-drug polymer in water in presence of TA, a successful Fluorescence Resonance Energy Transfer (FRET) signal was generated between the conjugated CPT (FRET donor) and conjugated CUR (FRET acceptor). Interestingly, these stable nano-assemblies showed a preferential breakdown and release of CPT in a tumor-relevant redox environment (in the presence of 50 mM glutathione), leading to the disappearance of the FRET signal. These nano-assemblies exhibited a successful cellular uptake by the cancer cells and an enhanced antiproliferative effect in comparison to the individual drugs in cancer cells (AsPC1 and SW480). Such promising in vitro results with a novel redox-responsive, dual-drug conjugated, FRET pair-based nanosized multimodal delivery vector can be highly useful as an advanced theranostic system towards effective cancer treatment.

## 1. Introduction

Among various cancer treatment strategies, chemotherapy is a commonly used and highly effective method [1,2,3]. To enhance the efficacy of chemotherapy against cancer, the co-delivery of multiple drugs (or therapeutic agents) has been implied [1,4,5,6,7,8]. Conventional mono- and combination chemotherapy are often ineffective due to high cytotoxicity, poor solubility of the hydrophobic chemotherapeutic drugs in a physiological environment, rapid blood clearance, nonspecific or uncontrolled bio-distribution of drugs, and undesired side effects to healthy cells or tissues [1,2,3]. To address these limitations, a nanomedicine-based delivery strategy involving nano-vehicles has been introduced [9,10,11,12,13]. However, co-delivery systems able to simultaneously carry and release hydrophobic anti-cancer drugs in cancer-relevant conditions are still limited. To overcome this issue, we propose to synthesize a fluorescence resonance-energy transfer (FRET) pair-based stimuli-responsive drug-drug conjugate using a hydrophilic PEG-based heterobifunctional crosslinker, where the chemotherapeutic drug curcumin (CUR) is PEGylated and then conjugated with another anti-cancer drug, camptothecin (CPT) via a redox-sensitive disulfide (-S-S-) linkage.

The FRET technique is considered one of the most efficient to be used in biological and material science applications [14,15,16]. Due to a high signal sensitivity and a specificity between donor and acceptor, a FRET-based donor-acceptor system is used not only to study interactions among various biomolecules but also to visualize drug release from the delivery system in a non-invasive and real-time manner [14,15,16,17,18,19]. In addition to the spectral overlap of the two fluorophores (i.e., FRET donor and acceptor), the FRET signal is also dependent on the separation distance between the FRET pair [14,15]. With the help of either physical entrapment inside a carrier or chemical linkage with the carrier, both the FRET donor and acceptor are brought together so that the fluorescence intensity of the donor will be quenched by the acceptor molecule to make a successful FRET pair [14,15]. Thus, the FRET signal will allow monitoring the in-situ release of any one of two probes (either donor or acceptor) in a stimulated condition due to the breakage of the stimuli-responsive bonds, leading to an increase of the donor-to-acceptor distance unfavorable for FRET. The aim to find such a specific FRET signal-based drug release from the stimuli-responsive carrier led us to choose two fluorescent anti-cancer drugs, camptothecin (CPT) and curcumin (CUR) as the FRET donor and acceptor, respectively. Here, we have conjugated both drugs to the same heterobifunctional water-soluble (hydrophilic) PEG chain, instead of performing a physical mixture of two drugs in a carrier or co-assembling two pro-drug systems to avoid the undesired release of the drugs from the delivery system before they reach the target site of action [7,20].

Furthermore, various tumor microenvironment-relevant physiopathological changes (e.g., pH, temperature and redox potential, enzymes) are found to be suitable for the development of stimuli-responsive nanoparticles as drug-delivery systems [21,22,23]. Among them, the tumor-relevant intracellular reductive environment has devoted enormous attention to the redox-responsive drug/gene-delivery system, which has disulfide (-S-S-) linkages in the backbone for the successful release of the therapeutic agent [12,24,25,26,27]. Based on this idea, we planned in this study to synthesize an amphiphilic macromolecule by conjugating a hydrophobic CPT to an amphiphilic PEGylated CUR through disulfide linkage (-S-S-), which would not only (i) allow the drug-drug conjugate to self-assemble in water due to hydrophobic interactions between the conjugated CPT and CUR molecules, but also (ii) make the conjugate sensitive to the elevated concentration of the reducing GSH, which triggers the release of the CPT at the desired site of action, followed by the destruction of the nano-assemblies. 

Both CPT (a naturally occurring alkaloid) and CUR (a polyphenol) are reported as potent chemotherapeutic agents with promising preclinical efficacy against many types of cancers [12,28,29,30,31,32]. The closed lactone ring and the C-20-OH group of CPT are responsible for binding to DNA topoisomerase I, leading to cell damage [12,28]. However, poor aqueous solubility (2.5 µg/mL), inactivation of the lactone ring at a physiological pH, the preferential binding of the carboxylate form (rather than closed lactone form) with the human serum albumin of blood leading to rapid clearance from the body, and the unwanted toxicity on healthy cells has limited the use of CPT as an anti-cancer drug [12,28,33,34,35]. CUR, a polyphenol with a low molecular weight and low or no intrinsic toxicity, is derived from the rhizome of the plant Curcuma longa [29,30,31,32]. It has been widely used not only in traditional Ayurvedic and Chinese medicine but also for a range of pharmacological applications (e.g., as an anti-inflammatory, anti-microbial, anti-oxidant, anti-parasitic, anti-mutagenic, or as a treatment for the Anti-Human Immunodeficiency virus,) that include cancer prevention and anti-cancer therapy [36,37,38]. Like CPT, the utilization of CUR in anti-cancer therapy suffers due to its low solubility in an aqueous solution (≈20 µg/mL), its rapid degradation at physiological pH, leading to low systemic bioavailability [39], and its poor pharmacokinetics [40]. 

To address this clinical need and to utilize the extraordinary anti-cancer properties of CPT and CUR, they have been conjugated to a water-soluble nanocarrier using their hydroxy (-OH) group [13,33,41,42,43,44,45]. The conjugation of CPT and CUR with the same hydrophilic biocompatible PEG polymer chain may enhance the therapeutic benefit by altering their drug pharmacokinetics and the accumulation of drugs in tumors due to an enhanced permeation and retention (EPR) effect [33,41,42,46,47,48]. The preparation of such a water-soluble drug-conjugated PEG-based polymer by converting the drug into an inactive but more stable prodrug form will allow it to revert to the pharmacologically active agent in presence of any biological stimulus [13,33,41,43,45]. Furthermore, tannic acid (TA), one of many naturally occurring dietary polyphenolic compounds derived from plant sources and a subset of hydrolyzable tannin congeners, has shown its excipient value in pharmaceutics [49,50,51,52]. Recently, TA has successfully been used as an additive molecule due to its high water solubility, low viscosity, high biocompatibility, crosslinked network-forming ability via hydrogen bonds, and successful binding ability to drug molecules via hydrophobic interactions [53,54,55]. Such properties of TA have allowed it to be used to improve the solubilization of various hydrophobic drugs (curcumin, paclitaxel, amphotericin B, rapamycin, or docetaxel) inside the drug delivery system for localized delivery through a parenteral route [49,51]. Considering its benefits, we have used TA as a biocompatible additive for dual drug-conjugated PEG-based polymers to generate the stable nano-formulations in water and thereby develop a possible FRET system between these two drug molecules CPT (FRET donor) and CUR (FRET acceptor). 

In this study, our objectives were (1) to synthesize and characterize disulfide (–S–S–) linked CPT-anchored PEGylated CUR, (2) to evaluate its self-assembly formation into nanostructures leading to the occurrence of a possible FRET in the presence and absence of the additive molecule TA, (3) to assess its redox-responsiveness in various GSH concentrations, and (4) to study its therapeutic potential and cellular uptake on various cancer cells in in vitro condition.

## 2. Materials and Methods

### 2.1. Materials

Camptothecin (CPT), 3-tritylsulfanylpropionic acid (TTPA), 4-dimethylamino pyridine (DMAP) and N-(3-dimethylaminopropyl)-N′-ethylcarbodiimide hydrochloride (EDC), triethylsilane (TESi), trifluoroacetic acid (TFA), curcumin (CUR), PEG-based heterobifunctional crosslinker, ortho-pyridyl disulfide (OPSS) polyethylene glycol (PEG5000) succinimidyl carboxymethyl ester (OPSS-PEG5000-SCM), glutathione (GSH), were purchased from Sigma Aldrich (St. Louis, MO, USA) and used without further purification. Dichloromethane (DCM), diethyl ether, dimethyl sulfoxide (DMSO), ethanol, and deuterated dimethyl sulfoxide (DMSO-d_6_) were obtained from Sigma Aldrich (St. Louis, MO, USA). MilliQ water (with a resistivity of 18.2 MΩ.cm at 25 °C) was collected using a Milli-Q Biopak (IQ 7000) water purification system from Merck (Kenilworth, NJ, USA). Dulbecco’s Modified Eagle Medium (DMEM) and Roswell Park Memorial Institute 1640 medium (RPMI 1640) was purchased from Fisher Scientific (Gibco, Billings, MT, USA). Vectashield^®^ mounting medium containing propidium iodide (PI) was purchased from Vector Laboratories (Burlingame, CA, USA). AsPC1 and SW480 human pancreatic and colon cancer cell lines were purchased from American Type Culture Collection (ATCC, Manassas, VA, USA). 

### 2.2. Synthesis of Thiolated Camptothecin

**Figure 1:**
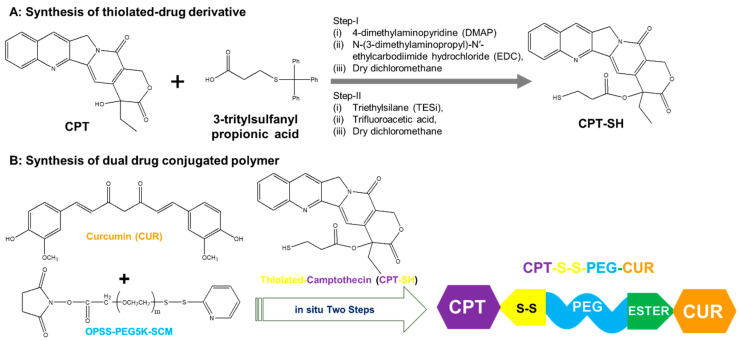
(**A**) Synthesis of the thiolated derivative of camptothecin (thio-camptothecin or CPT-SH) from CPT in two-step reactions; (**B**) Synthesis of CPT-S-S-PEG-CUR polymer through in situ two-step reactions using curcumin (CUR), CPT-SH and PEG-based heterobifunctional crosslinker, OPSS PEG succinimidyl carboxymethyl ester (OPSS-PEG-SCM), where m~114.

The thiol-derivative of camptothecin or thiolated camptothecin (CPT-SH) was synthesized (Figure 1A) following a previously reported procedure in two-step reactions [12]. In the first step, CPT (40 mg, 1 equivalent), 3-tritylsulfanylpropionic acid (40.01 mg, 1 equivalent), DMAP (24.21 mg, ~1.1 equivalents) and EDC (15.43 mg, ~1.1 equivalents) were mixed thoroughly in anhydrous 4 mL DCM and the reaction mixture was stirred overnight at 20 °C in dark surroundings. The reaction mixture was then filtered using Whatman cellulose filter paper to remove the solid part of the reaction mixture. After evaporation of the DCM solvent, the residue was redissolved in 4 mL of anhydrous DCM and left for stirring after the sequential addition of triethylsilane (0.5 mL) and trifluoroacetic acid (0.8 mL) for 2 h at 20 °C. Then DCM and TFA were removed from the reaction mixture by evaporation for 2 h, and the residual reaction mixture was redissolved in 500 μL DCM before being poured into an excess of cold diethyl ether (15 mL) to precipitate the final product, CPT-SH. The precipitated yellow powder was collected and dried under the vacuum hood. The infrared and ^1^H-NMR spectra (~3.1 mg in 0.5 mL DMSO-d_6_) of CPT-SH were recorded using a Nicolet AVATAR 380 Fourier-transform infrared (FTIR) spectrophotometer with a transmittance at mid-IR range (~400–4000 wavenumbers with 0.5 wavenumbers maximum resolution) and a Bruker Avance III-HD 400 MHz NMR spectrometer (based on residual proton resonance of the solvent as the internal standard), respectively (Supplementary Materials, Figures S1 and S2).

### 2.3. Synthesis of Amphiphilic Dual Drug Conjugated Polymer 

The amphiphilic dual drug conjugated polymer (CPT-S-S-PEG-CUR) was synthesized by conjugating curcumin (CUR) and a thiolated derivative of camptothecin (thio-camptothecin or CPT-SH) to the PEG-based heterobifunctional crosslinker, OPSS PEG succinimidyl carboxymethyl ester (OPSS-PEG-SCM), through in situ two-step reactions in DMSO (Figure 1B). Briefly, CUR solution (~1.2 equivalents, 4.42 mg in 1.2 mL DMSO) was added dropwise for 20–30 min to a freshly prepared solution of OPSS-PEG-SCM (1 equivalent, 50.0 mg in 1 mL DMSO) and left in the dark for 24 h for stirring at room temperature (~25 °C). The reaction mixture gradually turned yellow from a colorless solution. CPTSH (~1.2 equivalents, 5.23 mg in 0.3 mL DMSO) was then added in one shot to the reaction mixture, and it was stirred at room temperature in the dark. With time, the reaction mixture became a cloudy yellow solution. After nearly 36–40 h, the reaction mixture was transferred into a dialysis tubing (molecular weight cut-off of 3.5 kDa) and dialyzed first against DMSO (~100 mL) for ~3-4 h and then against distilled water (2 L) for 3 days at room temperature by changing the water twice per day to remove organic solvents and excess water-soluble reagents. Finally, the dialyzed solution was filtrated through a Whatman cellulose filter paper and freeze-dried using a Labconco freeze dryer (Kansas City, MO, USA). The final product, CPT-S-S-PEG-CUR, a yellow compound, was stored at −20 °C for long-term storage. 

The infrared spectra of CPT-S-S-PEG-CUR and OPSS-PEG-SCM were measured using a Nicolet AVATAR 380 Fourier-transform infrared (FTIR) spectrophotometer with transmittance at mid-IR range (~400–4000 wavenumbers with 0.5 wavenumbers maximum resolution). The ^1^H-NMR spectrum of CPT-S-S-PEG-CUR (~2.2 mg in 0.5 mL DMSO-d_6_) was recorded using the residual proton resonance of the solvent as the internal standard on a Bruker Avance III-HD 400 MHz NMR.

### 2.4. Absorbance and Fluorescence of the Conjugated and Free Drugs

The absorbance (Abs) and fluorescence (Flu) spectra of CPT-S-S-PEG-CUR at various concentrations (0.25, 0.5, 1.0, and 2.0 mg/mL) in the presence and absence of TA (5 mg/mL) in aqueous (Milli-Q water), in absence of TA (5 mg/mL) in aqueous (Milli-Q water) and organic medium (DMSO) were measured by using a Varioskan LUX spectrophotometer (Thermo Scientific Varioskan LUX Multimode Microplate Reader, Thermo Fisher Scientific, Waltham, MA, USA). The absorbance spectra were recorded between 300–700 nm. Fluorescence spectra were recorded at 5 nm bandwidth for CPT (λ_ex_ = 365 nm, λ_em_ = 390–700 nm), and CUR (λ_ex_ = 420 nm, λ_em_ = 440–700 nm). 

### 2.5. Quantification of Conjugated Drugs

Quantification of polymer conjugated drugs (CUR and CPT) was performed using a UV–Vis spectrophotometer (Thermo Scientific Varioskan LUX Multimode Microplate Reader). In brief, the CPT-S-S-PEG-CUR stock solution (10 mg/mL) in DMSO was first diluted 20 times using DMSO (10 µL aliquot of stock in 190 µL DMSO) to dilute the polymer solution further. Then, the diluted solution (0.5 mg/mL) was properly vortexed for 5 min before taking the absorbance spectra (300–800 nm) and absorbance value at a particular wavelength (λ_max_ = 425 nm for CUR and λ_max_ = 365 nm for CPT). The study was repeated in triplicate. A standard curve of CPT and CUR was plotted using absorbance at the same wavelengths (λ_max_ = 425 nm for CUR and λ_max_ = 365 nm for CPT) at various concentrations of the drug (0.035, 0.03, 0.025, 0.02, 0.015, 0.01, 0.005, 0.0025, 0.001, and 0.0005 mg/mL) in DMSO, which was then used to determine the concentration of the conjugated drugs (CPT and CUR). 

### 2.6. Hydrodynamic Size and Zeta Potential 

The hydrodynamic diameter (d_H_) and zeta potential of self-assembled nanostructures of CPT-S-S-PEG-CUR at various concentrations (0.25, 0.5, 1.0, and 2.0 mg/mL) in the presence and absence of TA (5 mg/mL) in an aqueous medium (Milli-Q water) were measured at 37 °C by using a Malvern Zetasizer Ultra instrument (Malvern, UK). 

### 2.7. Circular Dichroism (CD) Spectroscopy

The Circular dichroism (CD) spectra of our samples in a standard 1 cm x 1 cm cuvette was recorded from 163 to 700 nm using a Jasco815 CD Spectrometer. Polymer solutions (0.25–2.0 mg/mL in aqueous media) in the absence and presence of TA (5 mg/mL) were prepared and incubated at room temperature before CD measurement at 25 °C. Background spectra of the water were acquired and then subtracted from the sample spectra and smoothed before plotting.

### 2.8. Transmission Electron Microscopy Imaging

The morphology of the self-assembled nanostructures by CPT-S-S-PEG-CUR in the absence and presence of TA (5 mg/mL) was observed using FEI Tecnai Osiris (Scanning) Transmission Electron Microscope (TEM) operating at an accelerating voltage of 200 kV. One drop (3 µL) of the test polymer solutions (1 and 2 mg/mL) in the absence and presence of TA (5 mg/mL) were cast on a TEM carbon-coated copper grid (200 mesh size) and dried in a desiccator overnight before being imaged.

### 2.9. Redox-Sensitive CPT Release and Change in FRET Signal

The redox-responsive disintegration of CPT from CPT-S-S-PEG-CUR was studied in the presence of a biological reducing agent, glutathione (GSH), by monitoring the change of the FRET fluorescence intensity as well as normal fluorescence intensity of the CUR. For that, a stock solution of CPT-S-S-PEG-CUR (2 mg/mL) and glutathione solutions (100 mM and 100 µM) were prepared in Milli-Q water in the presence of TA (5 mg/mL). Then the 150 µL of 1 mg/mL test polymer (CPT-S-S-PEG-CUR) samples in the presence or absence of various glutathione concentrations (0, 10 µM, 10 mM, and 50 mM) were prepared as follows:

(i) No GSH: 75 μL of polymer (2 mg/mL) with 75 μL TA solution,
(ii) 10 μM GSH: 75 μL of polymer (2 mg/mL) with 60 μL TA solution and 15 μL glutathione solution (100 μM)
(iii) 10 mM GSH: 75 μL of polymer (2 mg/mL) with 60 μL TA solution and 15 μL glutathione solution (100 mM),
(iv) 50 mM GSH: 75 μL of polymer (2 mg/mL) with 75 μL glutathione solution (100 mM).

The fluorescence intensity of CPT and CUR for all the test samples was assessed at various time intervals (5 min, 15 min, 30 min, 1 h, 2 h, 4 h, 6 h, and 8 h) from the time of addition of the GSH solution using Varioskan LUX fluorometers. The data were acquired and processed with Thermo Scientific SkanIt Software for microplate readers. The samples were incubated in the dark at 37 °C inside the machine during the experiment.

### 2.10. Redox-Responsive Change in the Hydrodynamic Size of Self-Assemblies

The hydrodynamic diameter of CPT-S-S-PEG-CUR in the presence of TA in Milli-Q water incubated with various glutathione concentrations (0, 10 µM, 10 mM, and 50 mM) was measured at 37 °C by DLS. For that, a stock solution of CPT-S-S-PEG-CUR (2 mg/mL) and glutathione solutions (100 mM and 100 µM) was prepared in the Milli-Q water in the presence of TA (5 mg/mL). Then the 1200 µL of 1 mg/mL test polymer (CPT-S-S-PEG-CUR) samples in the presence or absence of various glutathione concentrations (0, 10 µM, 10 mM, and 50 mM) were prepared as follows:

(i) No GSH: 600 μL of polymer (2 mg/mL) with 600 μL TA solution,
(ii) 10 μM GSH: 600 μL of polymer (2 mg/mL) with 480 μL TA solution and 120 μL glutathione solution (100 μM),
(iii) 10 mM GSH: 600 μL of polymer (2 mg/mL) with 480 μL TA solution and 120 μL glutathione solution (100 mM),
(iv) 50 mM GSH: 600 μL of polymer (2 mg/mL) with 600 μL glutathione solution (100 mM).

The samples were incubated in a glass container in the dark at 37 °C. The change in the hydrodynamic diameter of the test samples was assessed at various time intervals (10 min, 1 h, 4 h, and 8 h) from the time of addition of the GSH solution.

### 2.11. In Vitro Cell Culture

AsPC1 and SW480 cells were grown as monolayers in 1% (*v*/*v*) L-glutamine containing Dulbecco’s Modified Eagle Medium (DMEM) and Roswell Park Memorial Institute 1640 medium (RPMI 1640), respectively, supplemented with 10% (*v*/*v*) fetal bovine serum and 1% (*v*/*v*) penicillin–streptomycin. Cells were cultured at 37 °C in a humid atmosphere with 5% carbon dioxide.

### 2.12. Confocal Microscopy

Using confocal microscopy, the cellular uptake of CPT-S-S-PEG-CUR in the presence and absence of TA was qualitatively assessed in comparison to other positive and negative controls. Both the cells (AsPC1 and SW480) were seeded on four-chambered (well) slides at a concentration of 50,000 cells per well in 0.5 mL media and left to attach and grow at 37 °C for 24 h. After that, the old media was aspirated, and the cells were then treated with CPT-S-S-PEG-CUR at various CUR-equivalent concentrations (5, 10, and 20 μM). Cells were treated with only CUR (5, 10, and 20 μM), only CPT (20 μM), and TA (equivalent to 10 μM CUR equivalent CPT-S-S-PEG-CUR formulation) solution as positive and negative controls, respectively, along with untreated cells. After 4 and 12 h of incubation at 37 °C, the treated and untreated cells were washed with phosphate-buffered saline (pH 7.4) twice (2 mL each time) and then the cells were allowed to be fixed using 2 mL methanol in the dark at room temperature. After ~15 min, methanol was removed completely and then the chamber was removed from the slide gently, according to the manufacturer’s protocol. Then the nuclei of the cells were stained with Vectashield^®^ mounting medium containing propidium iodide (PI), and the slide was covered with the cover slip, carefully removing the air bubbles. Finally, the treated cells were examined using a Nikon confocal ECLIPSE Ti2 microscope (Melville, NY, USA). Nuclear staining agent PI, intracellular CUR, and CPT were excited with the TRITC, FITC, and DAPI laser line (fixed channel) respectively.

### 2.13. Cell Proliferation Assay 

To evaluate the cell growth following treatment with free drugs (CUR and CPT) and dual-drug-conjugated amphiphilic polymer-drug formulation (CPT-S-S-PEG-CUR) at various concentrations, a standard MTT assay (a colorimetric assay for assessing cell metabolic activity) was performed. Briefly, AsPC1 and SW480 cells were seeded at a density of 5 000 cells per well in 96-well plates in 100 µL RPMI1640 and DMEM medium, respectively, and left to grow at 37 °C in a humid atmosphere with 5% carbon dioxide. The cells were allowed to attach for 24 h, and the medium was removed from the cells before being treated with the free drug or CPT-S-S-PEG-CUR polymer solutions (in the presence of TA) at various concentrations (for CPT group: a. 1.562 µM, b. 3.125 µM, c. 6.25 µM, d. 12.5 µM, e. 25 µM, and f. 50 µM, and for CUR group: a. 0.781 µM, b. 1.562 µM, c. 3.125 µM, d. 6.25 µM, e. 12.5 µM, f. 25 µM, and g. 50 µM), using a serial dilution. After 48 h of treatment, 20 µL of MTT solution (5 mg/mL *w*/*v* in PBS pH 7.4) were added in each well and further incubated for 3h at 37 °C to reduce MTT to formazan dye by the living cells. Then the medium was removed, and the dye was solubilized in 100 µL DMSO, before being quantified by spectrophotometry at 590 nm with a Varioskan LUX spectrophotometer (Thermo Scientific Varioskan LUX Multimode Microplate Reader). The percent proliferation in the drug and Nano-drug-treated cancer cells was calculated by normalizing the cells with no treatment, which was considered as 100%.

### 2.14. Bioinformatics Analyses

ADMET Analysis: BIOVIA Discovery Studio 2020 was used for the ADMET analysis of the test compound (Supplementary Materials, Figure S15), where m = 1 for simplification of the study. The ADMET descriptors were aqueous solubility, blood-brain barrier penetration, CYP2D6 binding, hepatotoxicity, intestinal absorption, and plasma protein binding. For further toxicity analysis, we used the TOPKAT plugin of BIOVIA Discovery Studio 2020 in the analyses where we screened the toxicity potential of our test compound against various parameters like aerobic biodegradability, AMES mutagenicity, developmental toxicity potential, mild vs moderate-severe ocular irritancy, mild vs moderate-severe skin irritancy, skin sensitization vs, and weight of evidence of rodent carcinogenicity.

Reverse Docking: To identify in silico drug targets, we used the reverse docking approach, where the high-throughput pharmacophore mapping method was used for the selection of potential drug targets. A data set on human protein targets was selected for mapping purposes, with 300 maximum generated conformations [56].

Construction, visualization, pathways and functional enrichment of protein interaction network (PIN): The details of all finally selected 54 probable targets were obtained from the reverse docking approaches. The selection criteria of the key targets of the test compound were based on a Z score (≥1). This final list of 54 proteins was used for the construction of PIN by using a Cytoscape STRING plugin on the bases of text mining, experiments, databases, co-expression, neighborhood, gene fusion, and co-occurrence with a medium confidence interaction score. The ClueGo plugin of Cytoscape was used for the further pathway-enrichment analysis of PIN according to the Kyoto Encyclopaedia of Genes and Genomes 2021 (KEGG) database [57,58], with a threshold of *p* < 0.05 based on a two-sided hypergeometric test and the Bonferroni correction. A redial layout was used to construct the PIN interactome.

## 3. Results

### 3.1. Molecular Characterization

We successfully synthesized the first thiolated CPT derivative (CPTSH) and amphiphilic polymer (CPT-S-S-PEG-CUR) through in situ two-step reactions (Figure 1). The successful synthesis of the CPTSH was proved by the corresponding FTIR and NMR peaks (Supplementary Materials, Figures S1 and S2) based on the previous report [12]. The synthesis of CPT-S-S-PEG-CUR was confirmed by the FTIR, ^1^H-NMR, and UV-Vis spectrum. The infrared spectrum of CPT-S-S-PEG-CUR (Supplementary Materials, Figure S3) revealed the presence of a new peak at 1650–1690 cm^−1^ (corresponding to an ester bond) and the absence of the distinct peak at 1740 cm^−1^ (corresponding to the succinimidyl carboxymethyl ester group) in comparison with that of the crosslinker, OPSS-PEG-SCM (Supplementary Materials, Figure S4). This proved the successful conjugation of curcumin (CUR) to PEG through ester bond formation. The conjugation of camptothecin (CPT) with the PEGylated CUR was also confirmed due to the presence of the peaks of CPT and PEG in the ^1^H-NMR spectrum (Supplementary Materials, Figure S5).

**Figure 2:**
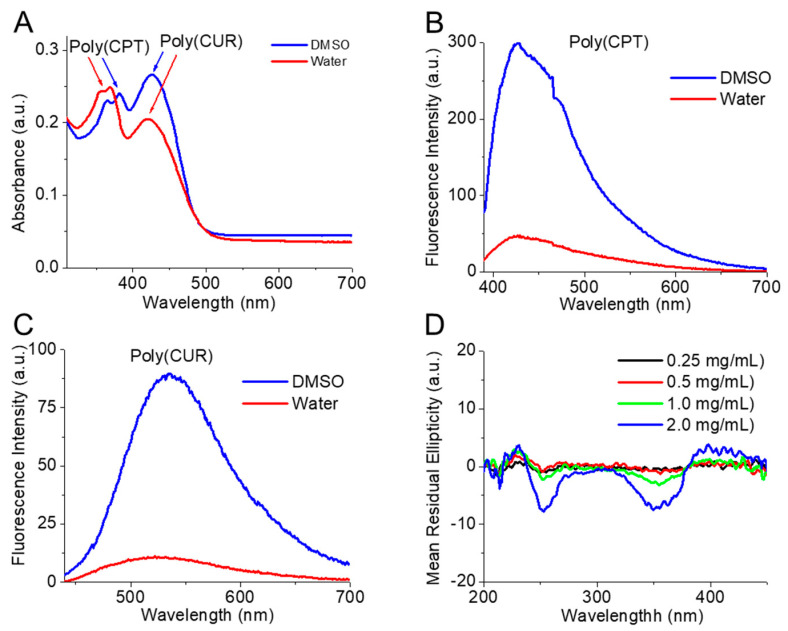
(**A**) Absorbance spectrum of the amphiphilic polymer (CPT-S-S-PEG-CUR) solution in DMSO and water (0.5 mg/mL, 50 µL). Fluorescence spectrum of (**B**) CPT (Ex = 365 nm, Em = 390–700 nm) and (**C**) CUR (Ex = 420 nm, Em = 440–700 nm) conjugated to the amphiphilic polymer (CPT-S-S-PEG-CUR) (10 mg/mL, 50 µL) in DMSO and water. (**D**) CD spectra of amphiphilic polymer (CPT-S-S-PEG-CUR) solution in water at various concentrations (0.25, 0.5, 1.0, and 2.0 mg/mL) in absence of TA.

The absorbance spectra of the purified dual drug-conjugated amphiphilic polymer CPT-S-S-PEG-CUR in water and DMSO showed the presence of both the peaks for CPT (~365 nm) and CUR (~425 nm) (Figure 2A), confirming that both the drugs are successfully conjugated to the water-soluble heterobifunctional PEG-based polymer. Successful conjugation will facilitate the use of such hydrophobic drugs (CPT and CUR) as hydrophilic pro-drug conjugates, which may further improve the bioavailability of these two drugs. Additionally, the characteristic fluorescence spectra of both the drugs without any change in the λ_max_ of the emission spectra in DMSO and water (Figure 2B,C) also proved the successful conjugation of CPT (~425 nm) and CUR (~525 nm) to the PEG chain without any change of their spectral properties. To investigate the packing of the chemically conjugated bioactive molecules within the polymeric backbone (or possible nano-aggregates), circular dichroism (CD) spectra of CPT-S-S-PEG-CUR solution at various concentrations (0.25, 0.5, 1.0 and 2.0 mg/mL) were recorded. Amphiphilic polymer thoroughly displayed distinctive CD signals in the absorption regions around 250 and 350 nm (Figure 2D). CD measures absorption differences between left and right circularly polarized light; consequently, the increasing CD signal with the concentration of the polymer suggested an increasing chiral packing of the conjugated drug moieties inside the polymeric backbone (or within the possible nano-assemblies) with negative chirality and left-handed helical arrangement.

### 3.2. Self-Assembly Behavior of CPT-S-S-PEG-CUR in Presence and Absence of TA

**Figure 3:**
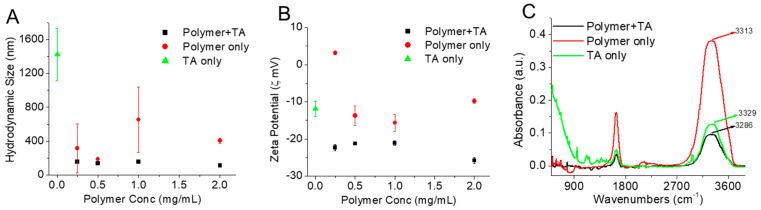
(**A**) Hydrodynamic size and (**B**) zeta potential of polymer (CPT-S-S-PEG-CUR) solutions in water at various concentrations (0.25, 0.5, 1.0, and 2.0 mg/mL) in presence and absence of TA (5 mg/mL) and only TA (5 mg/mL) at 37 °C (*n* = 3). (**C**) FTIR spectra of polymer (CPT-S-S-PEG-CUR) solution (1.0 mg/mL) in absence and presence of TA (5 mg/mL) and only TA solution (5 mg/mL). The spectra were acquired and plotted after water subtraction.

Smaller nano-assemblies in presence of TA: The formation of self-assembled nanostructures by CPT-S-S-PEG-CUR at various concentrations (0.25, 0.5, 1.0, and 2.0 mg/mL) in the absence and presence of TA (5 mg/mL) in an aqueous medium (Milli-Q water) was confirmed through the hydrodynamic diameter (d_H_) and zeta potential measurement at 37 °C. In the presence of TA, the amphiphile showed smaller and consistent (nearly homogeneous) nano-assemblies of a size around 100 nm at all the tested concentrations (0.25, 0.5, 1.0 and 2.0 mg/mL), in comparison with the absence of the TA and only TA solution (5 mg/mL) (Figure 3A). Zeta potential values of the amphiphile solution (0.25, 0.5, 1.0, and 2.0 mg/mL) in water also decreased and became consistent in the presence of TA around −20 to −25 mV, whereas the zeta potential values were comparatively higher for only the amphiphile and TA (Figure 3B). The consistently lower size with a high zeta potential of the amphiphile in the presence of TA indicated the formation of stronger and stable assemblies in comparison with the only amphiphile, which might be attributed to the probably stronger H-bonding between TA and the polymer along with the hydrophilic-lipophilic balance of these aggregates. Additionally, an increasing trend of turbidity for the Polymer-TA solution was observed with the increasing concentration of the polymer (from 0.25 mg/mL to 2.0 mg/mL), further indicating the possible formation of more compact colloidal particles (Supplementary Materials, Figure S6).

Stronger H-bonding in presence of TA. The FTIR characterization of only TA, only polymer (CPT-S-S-PEG-CUR), and the mixture of polymer (CPT-S-S-PEG-CUR)−TA solution in water was performed to prove the presence of possible H-bonding between the polymer and TA (Figure 3C). In the FTIR spectra, the presence of the broad and strong absorption bands at 3313 and 3329 cm^−1^ was observed due to the symmetrical stretching vibration of the hydroxyl (-OH) groups of PEG group (of the polymer) and TA, respectively. On the contrary, the -OH stretching peak of the Polymer−TA nano-assemblies shifted to a lower wavenumber of 3286 cm^−1^ due to the presence of hydrogen bonding, as it is reported that the formation of hydrogen bonding (intra- or intermolecular) reduces the force constants and thereby the vibrational frequencies (at lower wavenumbers) of the chemical bonds (i.e., hydroxyl groups) [59]. Such significant shifts of the absorption bands to lower wavenumbers for polymer-TA in comparison with their individual counterpart (either polymer or TA) indicate the formation of stronger H-bonding between amphiphilic polymer CPT-S-S-PEG-CUR and TA [59]. 

**Figure 4:**
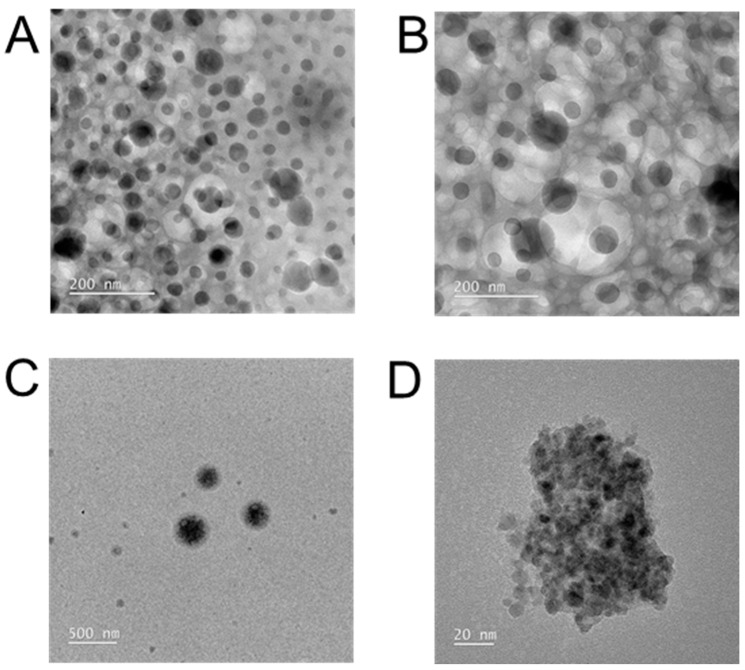
TEM images of the aqueous solutions of Polymer-TA mixture (**A**,**B**) and only polymer (**C**,**D**) at 1.0 mg/mL (**A**,**C**) and at 2.0 mg/mL (**B**,**D**).

**Scheme 1:**
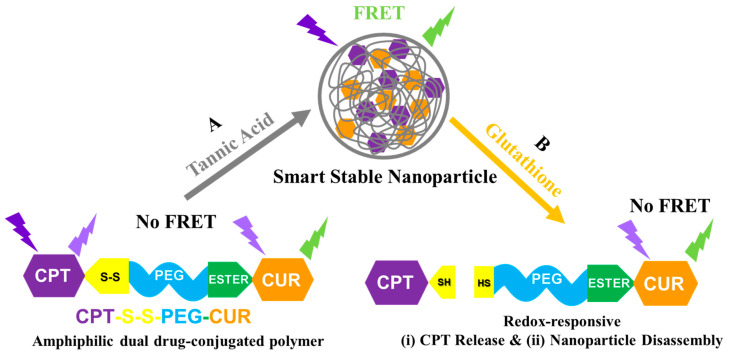
Cartoon representation of (**A**) nanoparticle formation: smart stable nano-assembly formation by amphiphilic polymer CPT-S-S-PEG-CUR in water in presence of TA; and (**B**) redox-responsiveness: GSH responsive CPT release, disassembly of nanoparticle and subsequent disappearance of FRET.

Formation of spherical nanoparticles in presence of TA: TEM images revealed the amphiphilic polymer CPT-S-S-PEG-CUR at various concentrations (1 and 2 mg/mL) in the presence of TA assembled into spherical-shaped nearly monodisperse core-shell nano-assemblies (polymeric micelles) with a size lower than 50 nm (Figure 4A,B). On the contrary, TEM images of only amphiphilic polymer CUR-PEG-SS-CPT also showed the formation of the core-shell nano-assemblies at the same concentrations, but these assemblies are much bigger (nearly 200 nm), irregular in shape, and polydisperse (Figure 4C,D). Thus, TA helped the dual-drug conjugated polymer to form stable and more compact core-shell nano-assemblies (Scheme 1). 

The trend of the nanoparticle formation (average size and size distribution) by the polymer in the absence of and presence of TA observed in TEM images is well supported by the DLS results (Figure 3A), though the size of polymeric nanoparticles obtained from the TEM images was found to be smaller than that observed with DLS. The DLS measurement is considered as the interference of the dispersant during hydrodynamic diameter measurement, whereas TEM measures the size of the sample after drying on the copper grid [60].

### 3.3. Occurrence of FRET in Presence of TA 

**Figure 5:**
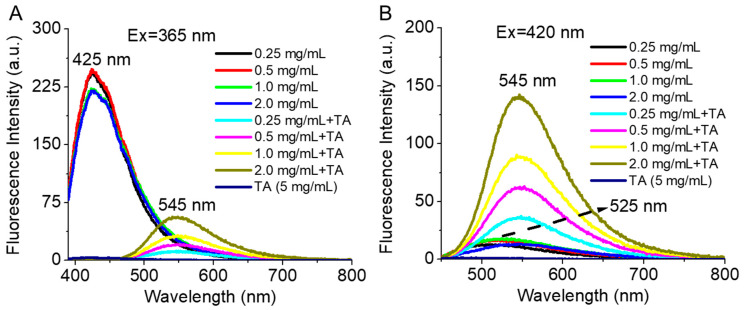
Fluorescence spectra of conjugated drugs (**A**) CPT (Ex = 365 nm) and (**B**) CUR (Ex = 420 nm) with the polymer CPT-S-S-PEG-CUR at various concentrations (0.25, 0.5, 1.0, and 2.0 mg/mL) in the absence and presence of TA (5 mg/mL).

The intense CPT fluorescence (Ex = 365 nm, the spectrum peak at 425 nm) of the amphiphilic polymer CPT-S-S-PEG-CUR was observed in the absence of TA. The fluorescence intensity of polymer-conjugated CPT was quenched in the presence of TA, and instead, CUR fluorescence spectra (the spectrum peak at 545 nm) appeared, indicating the occurrence of FRET between polymer-conjugated CPT and CUR in the presence of TA (Figure 5A). It was also observed that the conjugated CUR has a new emission peak at 545 nm in the presence of TA, instead of its usual emission peak at 525 nm in the absence of TA. The shift of the emission maxima of CUR in the presence of TA is inconsistent after the occurrence of the FRET. Even the FRET fluorescence intensity of the CUR in the presence of TA was found to be increased with the increment of the concentration of the polymer, indicating the occurrence of a better FRET at a higher concentration of the polymer (i.e., drug molecules). The formation of smaller-sized nano-assemblies in the presence of the TA (Figure 3A and Figure 4A,B) has helped both the drug molecules to come close enough for the FRET to occur. It was found that normal CUR fluorescence intensity (Ex = 420 nm) of the conjugate in the presence of TA also increased with the increment of the polymer concentration (Figure 5B), which might be attributed to the smaller nano-assembly formation with the increasing polymer concentration in the presence of the TA.

### 3.4. Reduction-Responsive Drug Release and Disintegration of Nanoparticles

The CPT is conjugated with the PEGylated curcumin via a disulfide (-S-S-) linkage, which is sensitive to the reducing agent GSH (Scheme 1B). Thus, the redox-responsiveness of these stable polymeric nanoparticles in the presence of TA was investigated by monitoring the changes in the FRET intensity (using fluorescence spectroscopy) and hydrodynamic sizes (using DLS measurements) in the absence and presence of various GSH concentrations (10 μM, 10 mM, and 50 mM, corresponding to reducing environments in the extracellular and intracellular compartments of normal cells and the intracellular compartment of tumor cells, respectively) at different time intervals.

**Figure 6:**
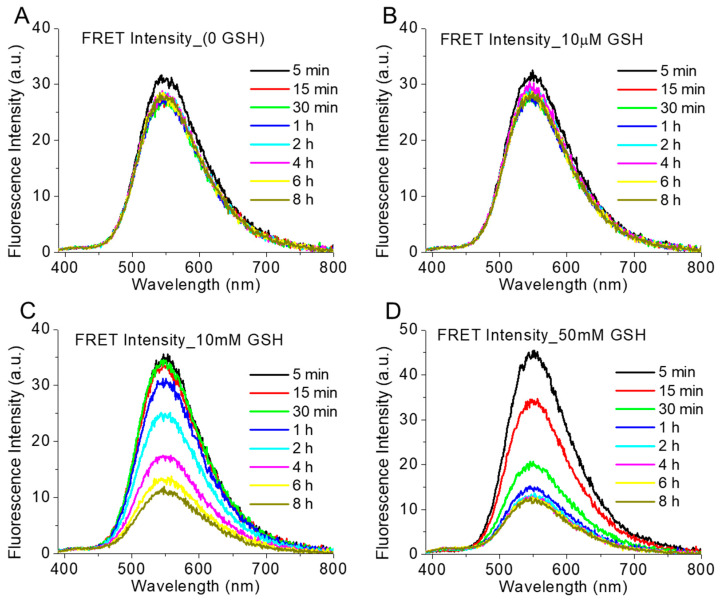
The intensity of FRET emission spectra (λ_ex_ = 365 nm, λ_em_ = 390–800 nm) of polymer (1 mg/mL)-TA (5 mg/mL) nanoparticle in presence of various GSH concentrations ((**A**) 0, (**B**) 10 µM, (**C**) 10 mM and (**D**) 50 mM) at 37 °C at various time intervals.

**Figure 7:**
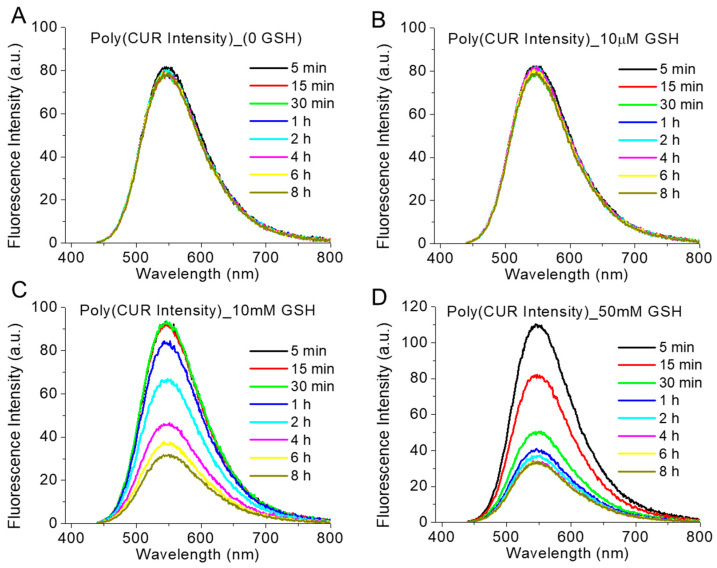
The intensity of CUR emission spectra (λ_ex_ = 420 nm, λ_em_ = 440–800 nm) of polymer (1 mg/mL)-TA (5 mg/mL) nanoparticle in presence of various GSH concentrations ((**A**) 0, (**B**) 10 µM, (**C**) 10 mM and (**D**) 50 mM) at 37 °C at various time intervals.

GSH-responsive FRET intensity change: We observed the reductive-responsive decrease of the FRET fluorescence intensity (λ_ex_ = 365 nm, λ_em_ = 390–800 nm) of polymer-TA nanoparticles with time in a comparatively higher reductive-mimicking medium containing 10 mM and 50 mM GSH, in comparison to that in the absence (non-redox condition) and in the presence of a 10 µM GSH concentration (extracellular condition) (Figure 6). Furthermore, in the presence of 50 mM GSH, FRET intensity was decreased more rapidly than that in the presence of the 10 mM (Figure 6C,D). In the presence of glutathione, as in a highly reductive environment, the CPT molecule was probably released from the nanoparticle due to the breakage of the disulphide linkage, making CPT unavailable for FRET with CUR and the concomitant reduction of FRET intensity. Similarly, we also observed the decrease in normal CUR fluorescence intensity (λ_ex_ = 420 nm, λ_em_ = 440–800 nm) with time in presence of 10 mM and 50 mM GSH concentrations in comparison to that in the absence (non-redox condition) and in the presence of a 10 µM GSH concentration (extracellular condition) (Figure 7). Loss of CUR fluorescence intensity further indicated the breakage of the nanoparticles with time in a comparatively higher reductive environment due to the release of the CPT from the nanoconjugates. Such a consistent decrease in fluorescence intensity (FRET and CUR intensity) for both the drugs (CPT and CUR), respectively, at a higher reductive environment proved the potential self-indicative release of the CPT inside cancer cell-specific conditions, making the strategy possible for non-invasive online monitoring of the drug release in in vivo condition.

**Figure 8:**
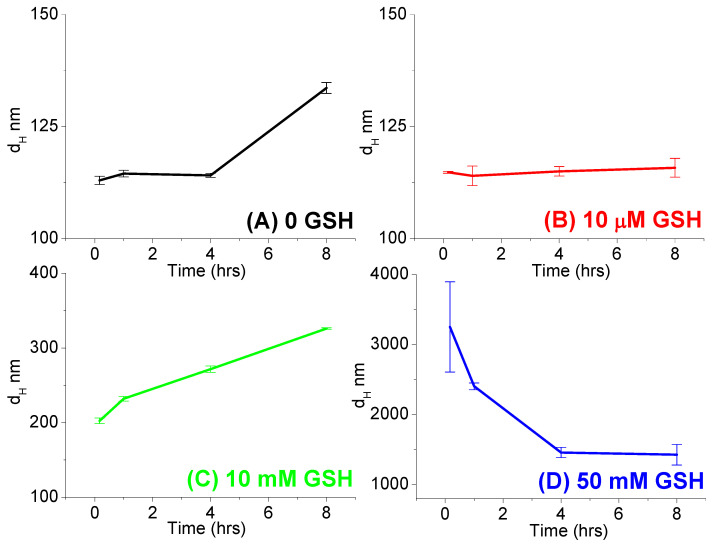
Average hydrodynamic sizes (d_H_ nm) of the polymer (1 mg/mL)-TA (5 mg/mL) nanoparticles in absence ((**A**) 0 GSH) and presence of various GSH concentrations ((**B**) 10 µM, (**C**) 10 mM and (**D**) 50 mM) at various time intervals (0.166 h, 1 h, 4 h, and 8 h) at 37 °C.

GSH-responsive disintegration of the nanoparticles: The redox-sensitive nature of polymer-TA nanoparticles was also proved by measuring the hydrodynamic size distribution of the nanoparticle at various GSH concentrations (0, 10 μM, 10 mM, and 50 mM) at various time intervals. It was observed that the size of the nanoparticles was unperturbed (nearly 100 nm) in the absence of GSH as well as in the presence of 10 µM GSH, indicating no breakage of the nanoparticles with time. Even the size of the nanoparticle increased gradually only up to ~350 nm in the presence of 10 mM GSH. On the contrary, the size of the nanoparticle increased very rapidly (within 1 h) to around ~3500–2500 nm in the presence of 50 mM GSH concentrations, which remained more than 1000 nm for up to 8 h (Figure 8) due to the GSH-responsive disintegration of the nanoparticles supporting the fluorescence-based studies (Figure 6 and Figure 7). Respective images of polymer-TA solution in the presence of various GSH concentrations (Supplementary Materials, Figure S7) also supported the same conclusion. Such GSH-sensitive changes in hydrodynamic size and fluorescence intensity indicated the GSH-responsive disintegration of these stable nanoparticles in tumor-relevant reductive conditions (50 mM GSH concentration), leading to the precipitation of the particles and confirming the potential of such nanoparticles for redox-responsive cancer therapy.

### 3.5. Cellular Uptake of the Polymer CPT-S-S-PEG-CUR

**Figure 9:**
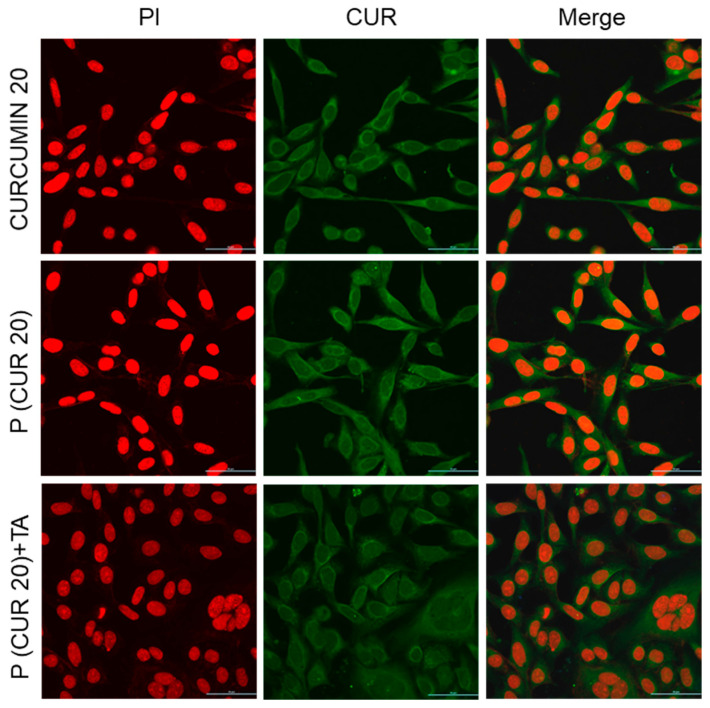
Cellular uptake of only-drug CUR and polymer CPT-S-S-PEG-CUR in absence and presence of TA by AsPC1 pancreatic cancer cell at 20 µM CUR-equivalent concentrations after 4 h of incubation (magnification: ×40, Zoom: 2.08, scale bar: 50 µm).

**Figure 10:**
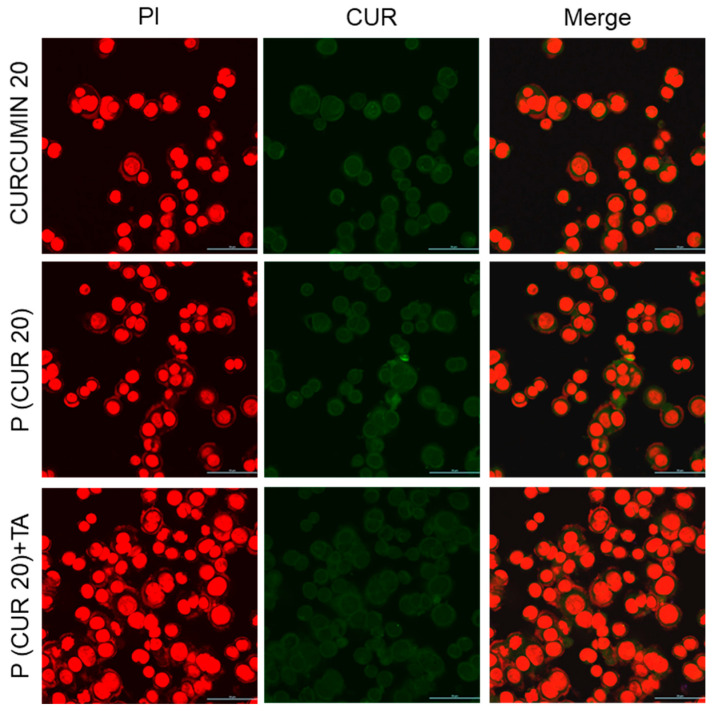
Cellular uptake of only-drug CUR and polymer CPT-S-S-PEG-CUR in absence and presence of TA by SW480 colon cancer cell at 20 µM CUR-equivalent concentrations after 4 h of incubation (magnification: ×40, Zoom: 2.08, scale bar: 50 µm).

For in vitro studies, two cancer cell lines, AsPC1 (pancreatic cancer) and SW480 (colorectal cancer) were chosen due to our previous successful work on the development of delivery systems and biomarker discovery for these cancer cells [30,61,62]. Our new strategy will allow us further to improve therapeutic response against these two cancers. The ability of polymer (in the presence and absence of TA) to be taken up by AsPC1 and SW480 cells following 4 h (and 12 h only for AsPC1) of incubation was assessed by confocal microscopy, using the fluorescence of the polymer-conjugated CUR drug. Confocal microscopy images (Figure 9 and Supplementary Materials, Figure S8 for AsPC1, Figure 10 and Supplementary Materials, Figure S9 for SW480) confirmed the successful cellular uptake of polymer-conjugated CUR (green fluorescence) in the presence and absence of TA at 20 and 10 µM CUR-equivalent concentrations for both the cell lines (AsPC1 and SW480). Nevertheless, we did not observe any distinct change in fluorescent intensity between cells treated with free (unconjugated) CUR and polymer-conjugated CUR (at 20 and 10 µM CUR-equivalent concentrations). After 4 h of incubation, CUR (or polymer) uptake appeared to be higher, followed by the treatment with the polymer at 20 µM CUR-equivalent concentrations than that treated with the polymer at 10 µM CUR-equivalent concentrations. Further, CUR (or polymer) uptake appeared to be even higher after 12 h of treatment with the polymer at 20 µM CUR-equivalent concentrations than that incubated for 4 h for AsPC1 cells, which might be attributed to the longer incubation time (Supplementary Materials, Figure S10). CUR (or polymer) was found to be localized in the cytoplasm of the cells. Fluorescence images of intracellular CPT were not presented as we were unable to observe a substantial amount of CPT fluorescence (either free or conjugated) inside both the cells up to 20 µM concentrations (not shown).

### 3.6. Antiproliferative Activity of Polymer CPT-S-S-PEG-CUR

**Figure 11:**
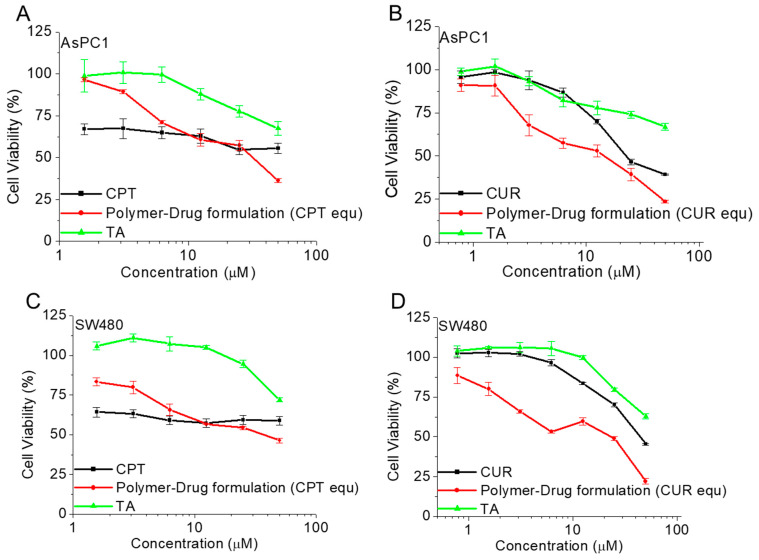
Cell viability (%) of only-drugs (CPT, CUR), and polymer-drug formulation (CPT-S-S-PEG-CUR) in presence of TA, and only TA on AsPC1 (**A**,**B**) and SW480 (**C**,**D**) cancer cells at various corresponding drug(CPT/CUR)-equivalent concentrations (for CPT group: a. 1.562 µM, b. 3.125 µM, c. 6.25 µM, d. 12.5 µM, e. 25 µM, and f. 50 µM and for CUR group: a. 0.781 µM, b. 1.562 µM, c. 3.125 µM, d. 6.25 µM, e. 12.5 µM, f. 25 µM, and g. 50 µM) after 48 h of incubation (*n* = 3).

The antiproliferative activity of the free drugs (CPT and CUR) and pro-drug polymer CPT-S-S-PEG-CUR (in presence of the TA) at various concentrations was tested on AsPC1 and SW480 cells for 48 h. Both cell lines showed an inhibition of cell proliferation in a dose-dependent manner. The obtained CUR and CPT treatment-dosing pattern was almost similar to that of the previously published studies [63,64]. For the CPT group, treatment with pro-drug polymer CPT-S-S-PEG-CUR showed comparatively higher antiproliferative activity in comparison to that treated with free CPT from a 50 µM concentration onwards on both the AsPC1 and SW480 cells (Figure 11A,C, and Figures S12 and S14). Similarly, for the CUR group, treatment with the pro-drug polymer CPT-S-S-PEG-CUR showed a comparatively higher antiproliferative activity from the very early screening concentrations (at 3.125 µM for AsPC1 and at 1.562 µM for SW480), in comparison with that treated with only CUR (Figure 11B,D, Figures S11 and S13). That trend of increased antiproliferative activity remained similar up to 12.5 µM for AsPC1 and 50 µM for SW480. Such an enhanced anti-proliferative effect of the water-soluble drug-conjugated polymer would be advantageous for in vivo study, rather than the hydrophobic free drugs. For both the groups (CPT and CUR) on both the cell lines (AsPC1 and SW480), the TA remained highly cell-viable up to a very high drug-equivalent concentration, justifying its use as an additive molecule for such polymer-TA formulation.

### 3.7. Bioinformatics Analyses

ADMET Analysis: The test compound was screened on the bases of ADMET parameters by using BIOVIA Discovery Studio 2020. In these analyses, the tested compound was unable to cross the blood-brain barrier, and showed no CYP2D6 binding, non-hepatotoxicity, low intestinal absorption, and negative plasma protein binding. Apart from that topkat analysis, this query compound is aerobically biodegradable, with no Ames mutagenicity, mild ocular irritability, mild skin irritation, no skin sensitizing, and no rodent carcinogenicity. 

Reverse Docking: Through the reverse docking approach, around 300 targets were retrieved against the query compound. These 300 targets were further screened on the basis of the Z-Score. The targets with a Z-score equal to or more than 1, were selected as probable targets of the test compound. In this method of screening, a total of 54 key targets were selected out of 300 targets as probable interactors of our query compound (Table 1).

**Figure 12:**
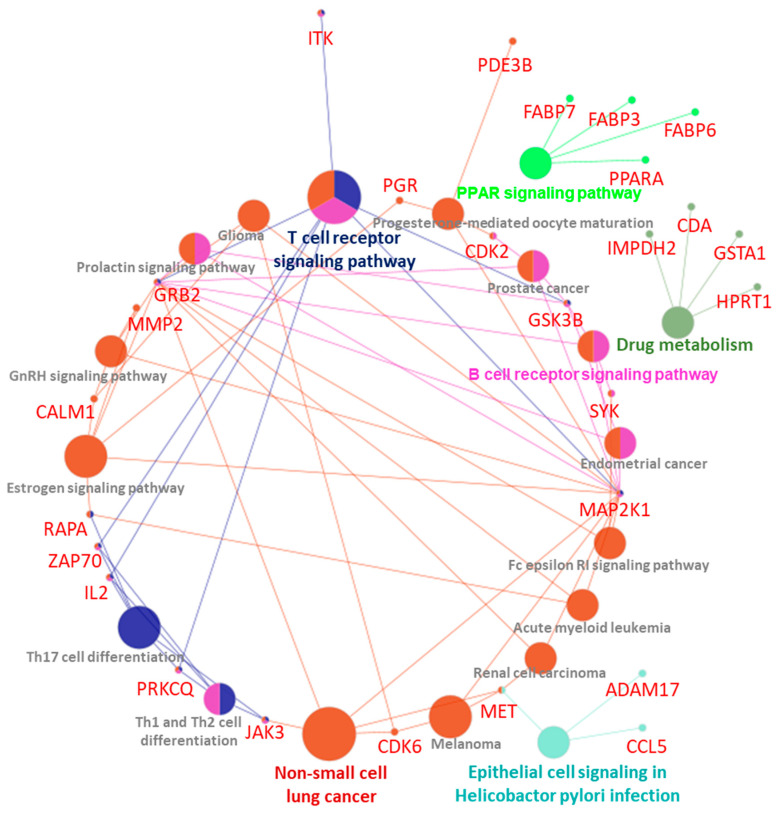
Interactome and pathways enrichment analysis of the test compound.

Interactome and pathways enrichment analysis: PIN and pathways enrichment analyses of the key 54 protein targets of the test compound were used. A ClueGo-KEGG pathway plugin was used to enrich the pathways and the function of the target proteins. A total of 19 pathways were enriched in this study, and these proteins were found to follow various important pathways like epithelial cell signaling in a helicobacter pylori infection, endometrial cancer, Fc epsilon RI signaling pathway, prolactin signaling pathway, renal cell carcinoma, melanoma, acute myeloid leukemia, drug metabolism, PPAR signaling pathway, Th1 and Th2 cell differentiation, B cell receptor signaling pathway, prostate cancer, GnRH signaling pathway, progesterone-mediated oocyte maturation, glioma, Th17 cell differentiation, non-small cell lung cancer, estrogen signaling pathway and T cell receptor signaling pathway (Figure 12).

## 4. Discussion

In this proof-of-principle study, a dual chemotherapeutic drug-conjugated amphiphilic polymer, disulfide-linked CPT-bearing PEGylated CUR (CPT-S-S-PEG-CUR), has been successfully synthesized. To make such a construct, two chemotherapeutic drugs, CUR and thiolated CPT, were conjugated sequentially (in situ two-step reactions) with the same hydrophilic PEG chain (MW 5000) through ester and disulphide linkage, respectively. To our knowledge, this is the first report of CPT-conjugated PEGylated CUR where the amphiphilic polymer was able to spontaneously self-assemble in the presence of a physical crosslinker TA into anionic, comparatively smaller-sized (~100 nm) stable nanoparticles in water (Figure 3A,B and Figure 4A,B) due to the formation of the stronger H-bonds between TA and an amphiphilic polymer chain (more specifically with the PEG chain). The polymer alone (in absence of TA) was unable to form such stable, smaller-sized particles with a narrow size distribution. Such a strategy of preparing a stable nano-formulation with more than one drug, employing combination therapy, could be a useful and better alternative than TA-assisted nano-formulation containing only one drug, as in previous reports [49,51]. During such nanoparticle formation by the amphiphilic polymer, lipophilic drugs may form the hydrophobic core of the nanoparticle. Stable nanostructure formation by the amphiphilic polymer in the presence of TA in water, due to the hydrophobic–hydrophilic balance and stronger H-bonding, may enhance the stability of this nanostructured formulation in the biological milieu [13,65,66]. The generation of induced negative chirality and left-handed helical arrangement of the conjugated drug molecules in water with the increasing concentration of polymer solution might be attributed to the probable secondary structure (local spatial arrangement) of the polymer chain. 

Interestingly, the successful spectral overlap between these two anti-cancer fluorescent drugs (CPT and CUR) and the formation of a stable nano-assembly in the presence of TA in an aqueous media allowed these drugs to come close enough to produce a FRET signal, where CPT and CUR act as FRET donors and acceptors, respectively (Figure 5A). Additionally, self-assembly (or nanoparticle) formation also enhanced the fluorescence intensity of the polymer-conjugated CUR in the presence of TA in comparison to that of the polymer-conjugated CUR in the absence of the TA (Figure 5B). Such enhancement of conjugated CUR fluorescence intensity in the presence of TA indicated a better packaging of the hydrophobic drug molecule inside the nanoparticle’s core. Furthermore, a successful FRET signal between chemically conjugated chemotherapeutic drugs (CPT and CUR) to the same polymer may well be an effective strategy instead of physically encapsulating them within the cationic nanoparticle or co-assembling two pro-drug lipid carriers [7,20]. The chemical conjugation of both drugs with the same carrier may minimize (i) the undesired release of the drug molecules before reaching the site of action for drug-encapsulated nanoparticles and (ii) the optimization process for co-assembled nanoparticle formation for an in vivo study [7,20]. 

This self-assembled nano-assembly not only exhibited successful cellular uptake in the presence and absence of TA, but also showed an enhanced antiproliferative effect in various cancer cells (AsPC1 and SW480) in comparison to the individual drugs. The presence of a reducing biothiol was found to be different in various subcellular regions of the cells, such as glutathione (GSH) presents in different amounts in intracellular (~10 mM) and extracellular (<10 μM) compartments of living cells, and nearly 4-fold higher in the intracellular condition of cancer than in normal tissues. Such a concentration gradient makes the GSH an interesting stimulus to release the chemically conjugated drug attached through the disulphide (-S-S-) linkages with drug delivery systems in cancer cell-specific redox-responsive conditions [12,24,25,26,27]. Interestingly, TA-assisted stable nano-assemblies showed a preferential cleavage of the nanoparticle, forming inconsistent and bigger particles with the concomitant release of CPT in a tumor-relevant redox environment (50 mM GSH concentration), leading to the disappearance of the FRET signal. Thus, such a GSH-responsive FRET pair-based stable nano-formulation will not only be highly effective for passively targeted cancer treatment but will also be helpful to monitor the drug release in a non-invasive manner.

Additionally, computational biology studies reflect the good draggability characteristics of the test compound. Most pharmacokinetics and toxicological parameters fall in preferred regions along with the broad therapeutic potential. Network analysis demonstrated important CPT-S-S-PEG-CUR-associated proteins, which are enriched with various cancers, such as endometrial cancer, renal cell carcinoma, melanoma, acute myeloid leukemia, prostate cancer, and non-small-cell lung cancer pathways (Figure 12). This will guide us for the future development of this conjugate. 

These promising preliminary in vitro results will allow us to test this compound and strategy further in vitro and in vivo conditions to validate the FRET signal between these two drugs, along with other sets of anti-cancer fluorescent drugs. In our future investigations, we will also optimize the amount of TA, the length of the PEG chain of the crosslinker, and various other drugs (acting in synergy) to make this multimodal theranostic strategy more compatible, versatile, and robust for treating cancer chemo and multimodal therapies [67,68,69]. 

## 5. Conclusions

In summary, a dual chemotherapeutic drug-conjugated amphiphilic polymer, CPT-S-S-PEG-CUR, was synthesized by conjugating CPT with PEGylated CUR through disulfide (-S-S-) linkage using an in situ two-step reaction strategy. CPT-S-S-PEG-CUR was able to spontaneously form smaller-sized (~100 nm) stable anionic nano-assemblies in water only in presence of TA due to the presence of the stronger H-bonds between TA and amphiphilic polymer, allowing two drug molecules to come close enough to produce a FRET signal, where CPT acts as a FRET donor and CUR as a FRET acceptor. Conjugated drug molecules demonstrated induced negative chirality and a left-handed helical arrangement with the increasing concentration of polymer. Such TA-assisted stable nano-assemblies were found to be disintegrated in a tumor-relevant redox environment (50 mM GSH concentration) due to breakage of the disulfide linkage, leading to the disappearance of the FRET signal along with the concomitant release of the CPT drug. These nano-assemblies not only exhibited successful cellular uptake in the presence and absence of TA, but also showed an enhanced antiproliferative effect in various cancer cells (AsPC1 and SW480) in comparison to the individual drugs. Thus, promising preliminary in vitro results with such a GSH-responsive FRET pair-based stable nano-formulation consisting of a CPT-S-S-PEG-CUR polymer and TA can be highly effective for passively targeted cancer treatment and monitoring the drug release from the carrier in in vitro and in vivo conditions in a non-invasive manner. 

## Figures and Tables

**Table 1:** Functional and pathways enrichment analysis of key proteins of test compound.

GOTerm	Ontology Source	Nr. Genes	Associated Genes Found
Epithelial cell signaling in Helicobacter pylori infection	KEGG_10.03.2021	3	[ADAM17, CCL5, MET]
Endometrial cancer	KEGG_10.03.2021	3	[GRB2, GSK3B, MAP2K1]
Fc epsilon RI signaling pathway	KEGG_10.03.2021	3	[GRB2, MAP2K1, SYK]
Prolactin signaling pathway	KEGG_10.03.2021	3	[GRB2, GSK3B, MAP2K1]
Renal cell carcinoma	KEGG_10.03.2021	3	[GRB2, MAP2K1, MET]
Melanoma	KEGG_10.03.2021	3	[CDK6, MAP2K1, MET]
Acute myeloid leukemia	KEGG_10.03.2021	3	[GRB2, MAP2K1, RARA]
Drug metabolism	KEGG_10.03.2021	4	[CDA, GSTA1, HPRT1, IMPDH2]
PPAR signaling pathway	KEGG_10.03.2021	4	[FABP3, FABP6, FABP7, PPARA]
Th1 and Th2 cell differentiation	KEGG_10.03.2021	4	[IL2, JAK3, PRKCQ, ZAP70]
B cell receptor signaling pathway	KEGG_10.03.2021	4	[GRB2, GSK3B, MAP2K1, SYK]
Prostate cancer	KEGG_10.03.2021	4	[CDK2, GRB2, GSK3B, MAP2K1]
GnRH signaling pathway	KEGG_10.03.2021	4	[CALM1, GRB2, MAP2K1, MMP2]
Progesterone-mediated oocyte maturation	KEGG_10.03.2021	4	[CDK2, MAP2K1, PDE3B, PGR]
Glioma	KEGG_10.03.2021	4	[CALM1, CDK6, GRB2, MAP2K1]
Th17 cell differentiation	KEGG_10.03.2021	5	[IL2, JAK3, PRKCQ, RARA, ZAP70]
Non-small cell lung cancer	KEGG_10.03.2021	5	[CDK6, GRB2, JAK3, MAP2K1, MET]
Estrogen signaling pathway	KEGG_10.03.2021	6	[CALM1, GRB2, MAP2K1, MMP2, PGR, RARA]
T cell receptor signaling pathway	KEGG_10.03.2021	7	[GRB2, GSK3B, IL2, ITK, MAP2K1, PRKCQ, ZAP70]

## Data Availability

Data are contained within the article. The data presented in this study are available on request from the corresponding authors.

## Supplementary Materials

The following supporting information can be downloaded at: https://www.mdpi.com/article/10.3390/pharmaceutics15051326/s1, Figure S1: FTIR spectra of CPTSH; Figure S2: ^1^H-NMR spectrum of CPTSH (in DMSO-d6, 400 MHz); Figure S3: FTIR of dual drug (CPT and CUR) conjugated PEG-based polymer, CPT-S-S-PEG-CUR; Figure S4: FTIR spectra of starting material OPSS-PEG-SCM; Figure S5: ^1^H-NMR spectrum of dual drug (CPT and CUR) conjugated PEG-based polymer CPT-S-S-PEG-CUR (in DMSO-d6, 400 MHz); Figure S6: Turbidity (wavelength = 600 nm) of only TA solution (5 mg/mL) and CPT-S-S-PEG-CUR solution (0.25, 0.5, 1.0, and 2.0 mg/mL) in water in absence and presence of TA (5 mg/mL); Figure S7: Photographs of polymer (1 mg/mL)-TA nano-assemblies in absence and presence of various GSH concentration (10 μM, 10 mM, and 50 mM) in water at 37 °C; Figure S8: Cellular uptake of only drug CUR, and polymer CUR-PEG-S-S-CPT in absence and presence of TA by AsPC1 pancreatic cancer at 10μM CUR-equivalent concentrations after 4 h of incubation (magnification: ×40, Zoom: 2.08, scale bar: 50 μm); Figure S9: Cellular uptake of only drug CUR, and polymer CUR-PEG-S-S-CPT in absence and presence of TA by SW480 colon cancer cell at 10 μM CUR-equivalent concentrations after 4 h of incubation (magnification: ×40, Zoom: 2.08, scale bar: 50 μm); Figure S10: Cellular uptake of only drug CUR, and polymer CUR-PEG-S-S-CPT in absence and presence of TA by AsPC1 pancreatic cancer at 20 μM CUR-equivalent concentrations after 12 h of incubation (magnification: ×40, scale bar: 100 μm); Figure S11: Images of the AsPC1 cells treated with the CUR, Polymer (CUR equivalent) + TA, and TA (Polymer + TA equivalent) after 48 h; Figure S12: Images of the AsPC1 cells treated with the CPT, Polymer (CPT equivalent) +TA, and TA (Polymer + TA equivalent) after 48 h; Figure S13: Images of the SW480 cells treated with the CUR, Polymer (CUR equivalent) + TA, and TA (Polymer + TA equivalent) after 48 h; Figure S14: Images of the SW480 cells treated with the CPT, Polymer (CPT equivalent) +TA, and TA (Polymer + TA equivalent) after 48 h; Figure S15: 2D chemical representative diagram (A) and physical properties (B) of test compound.

## References

[1] Fan W., Yung B., Huang P., Chen X. (2017). Nanotechnology for Multimodal Synergistic Cancer Therapy. Chem. Rev..

[2] Sun W., Sanderson P.E., Zheng W. (2016). Drug combination therapy increases successful drug repositioning. Drug Discov. Today.

[3] Zhao G., Sun Y., Dong X. (2020). Zwitterionic Polymer Micelles with Dual Conjugation of Doxorubicin and Curcumin: Synergistically Enhanced Efficacy against Multidrug-Resistant Tumor Cells. Langmuir.

[4] Hu M., Huang P., Wang Y., Su Y., Zhou L., Zhu X., Yan D. (2015). Synergistic Combination Chemotherapy of Camptothecin and Floxuridine through Self-Assembly of Amphiphilic Drug-Drug Conjugate. Bioconjug. Chem..

[5] Huang P., Wang D., Su Y., Huang W., Zhou Y., Cui D., Zhu X., Yan D. (2014). Combination of small molecule prodrug and nanodrug delivery: Amphiphilic drug-drug conjugate for cancer therapy. J. Am. Chem. Soc..

[6] Li Y., Lin J., Ma J., Song L., Lin H., Tang B., Chen D., Su G., Ye S., Zhu X. (2017). Methotrexate-Camptothecin Prodrug Nanoassemblies as a Versatile Nanoplatform for Biomodal Imaging-Guided Self-Active Targeted and Synergistic Chemotherapy. ACS Appl. Mater. Interfaces.

[7] Xiao B., Si X., Han M.K., Viennois E., Zhang M., Merlin D. (2015). Co-delivery of camptothecin and curcumin by cationic polymeric nanoparticles for synergistic colon cancer combination chemotherapy. J. Mater. Chem. B.

[8] Li Y., Thambi T., Lee D.S. (2018). Co-Delivery of Drugs and Genes Using Polymeric Nanoparticles for Synergistic Cancer Therapeutic Effects. Adv. Healthc. Mater.

[9] Su Z., Dong S., Zhao S.C., Liu K., Tan Y., Jiang X., Assaraf Y.G., Qin B., Chen Z.S., Zou C. (2021). Novel nanomedicines to overcome cancer multidrug resistance. Drug Resist. Updat..

[10] Laskar P., Dey J., Banik P., Mandal M., Ghosh S.K. (2017). In Vitro Drug and Gene Delivery Using Random Cationic Copolymers Forming Stable and pH-Sensitive Polymersomes. Macromol. Biosci..

[11] Chauhan D.S., Dhasmana A., Laskar P., Prasad R., Jain N.K., Srivastava R., Jaggi M., Chauhan S.C., Yallapu M.M. (2021). Nanotechnology synergized immunoengineering for cancer. Eur. J. Pharm. Biopharm..

[12] Laskar P., Somani S., Campbell S.J., Mullin M., Keating P., Tate R.J., Irving C., Leung H.Y., Dufes C. (2019). Camptothecin-based dendrimersomes for gene delivery and redox-responsive drug delivery to cancer cells. Nanoscale.

[13] Cheetham A.G., Zhang P., Lin Y.A., Lock L.L., Cui H. (2013). Supramolecular nanostructures formed by anticancer drug assembly. J. Am. Chem. Soc..

[14] Chen T., He B., Tao J., He Y., Deng H., Wang X., Zheng Y. (2019). Application of Forster Resonance Energy Transfer (FRET) technique to elucidate intracellular and In Vivo biofate of nanomedicines. Adv. Drug. Deliv. Rev..

[15] Nhien P.Q., Chou W.L., Cuc T.T.K., Khang T.M., Wu C.H., Thirumalaivasan N., Hue B.T.B., Wu J.I., Wu S.P., Lin H.C. (2020). Multi-Stimuli Responsive FRET Processes of Bifluorophoric AIEgens in an Amphiphilic Copolymer and Its Application to Cyanide Detection in Aqueous Media. ACS Appl. Mater. Interfaces.

[16] Shi L., De Paoli V., Rosenzweig N., Rosenzweig Z. (2006). Synthesis and application of quantum dots FRET-based protease sensors. J. Am. Chem. Soc..

[17] Dong Z., Bi Y., Cui H., Wang Y., Wang C., Li Y., Jin H., Wang C. (2019). AIE Supramolecular Assembly with FRET Effect for Visualizing Drug Delivery. ACS Appl. Mater. Interfaces.

[18] Taemaitree F., Fortuni B., Koseki Y., Fron E., Rocha S., Hofkens J., Uji I.H., Inose T., Kasai H. (2020). FRET-based intracellular investigation of nanoprodrugs toward highly efficient anticancer drug delivery. Nanoscale.

[19] Zhang X., Xiong J., Wang K., Yu H., Sun B., Ye H., Zhao Z., Wang N., Wang Y., Zhang S. (2021). Erythrocyte membrane-camouflaged carrier-free nanoassembly of FRET photosensitizer pairs with high therapeutic efficiency and high security for programmed cancer synergistic phototherapy. Bioact. Mater..

[20] Li Y., Zhu J., Kang T., Chen Y., Liu Y., Huang Y., Luo Y., Huang M., Gou M. (2018). Co-assembling FRET nanomedicine with self-indicating drug release. Chem. Commun..

[21] Mi P. (2020). Stimuli-responsive nanocarriers for drug delivery, tumor imaging, therapy and theranostics. Theranostics.

[22] Mura S., Nicolas J., Couvreur P. (2013). Stimuli-responsive nanocarriers for drug delivery. Nat. Mater..

[23] Laskar P., Dey J., Ghosh S.K. (2016). Evaluation of zwitterionic polymersomes spontaneously formed by pH-sensitive and biocompatible PEG based random copolymers as drug delivery systems. Colloids Surf. B Biointerfaces.

[24] Laskar P., Dey J., Ghosh S.K. (2017). Spontaneously formed redox- and pH-sensitive polymersomes by mPEG based cytocompatible random copolymers. J. Colloid Interface Sci..

[25] Laskar P., Dufes C. (2021). Emergence of cationic polyamine dendrimersomes: Design, stimuli sensitivity and potential biomedical applications. Nanoscale Adv..

[26] Laskar P., Somani S., Altwaijry N., Mullin M., Bowering D., Warzecha M., Keating P., Tate R.J., Leung H.Y., Dufes C. (2018). Redox-sensitive, cholesterol-bearing PEGylated poly(propylene imine)-based dendrimersomes for drug and gene delivery to cancer cells. Nanoscale.

[27] Laskar P., Somani S., Mullin M., Tate R.J., Warzecha M., Bowering D., Keating P., Irving C., Leung H.Y., Dufes C. (2021). Octadecyl chain-bearing PEGylated poly(propyleneimine)-based dendrimersomes: Physicochemical studies, redox-responsiveness, DNA condensation, cytotoxicity and gene delivery to cancer cells. Biomater. Sci..

[28] Laskar P., Samanta S., Ghosh S.K., Dey J. (2014). In vitro evaluation of pH-sensitive cholesterol-containing stable polymeric micelles for delivery of camptothecin. J. Colloid Interface Sci..

[29] Batra H., Pawar S., Bahl D. (2019). Curcumin in combination with anti-cancer drugs: A nanomedicine review. Pharm. Res..

[30] Khan S., Setua S., Kumari S., Dan N., Massey A., Hafeez B.B., Yallapu M.M., Stiles Z.E., Alabkaa A., Yue J. (2019). Superparamagnetic iron oxide nanoparticles of curcumin enhance gemcitabine therapeutic response in pancreatic cancer. Biomaterials.

[31] Su P., Yang Y., Wang G., Chen X., Ju Y. (2018). Curcumin attenuates resistance to irinotecan via induction of apoptosis of cancer stem cells in chemoresistant colon cancer cells. Int. J. Oncol..

[32] Tan B.L., Norhaizan M.E. (2019). Curcumin Combination Chemotherapy: The Implication and Efficacy in Cancer. Molecules.

[33] Fleming A.B., Haverstick K., Saltzman W.M. (2004). In vitro cytotoxicity and in vivo distribution after direct delivery of PEG-camptothecin conjugates to the rat brain. Bioconjug. Chem..

[34] Omar R., Bardoogo Y.L., Corem-Salkmon E., Mizrahi B. (2017). Amphiphilic star PEG-Camptothecin conjugates for intracellular targeting. J. Control. Release.

[35] Guo Z., Lin L., Hao K., Wang D., Liu F., Sun P., Yu H., Tang Z., Chen M., Tian H. (2020). Helix Self-Assembly Behavior of Amino Acid-Modified Camptothecin Prodrugs and Its Antitumor Effect. Acs Appl. Mater. Interfaces.

[36] Yallapu M.M., Jaggi M., Chauhan S.C. (2010). beta-Cyclodextrin-curcumin self-assembly enhances curcumin delivery in prostate cancer cells. Colloids Surf. B Biointerfaces.

[37] Yallapu M.M., Othman S.F., Curtis E.T., Bauer N.A., Chauhan N., Kumar D., Jaggi M., Chauhan S.C. (2012). Curcumin-loaded magnetic nanoparticles for breast cancer therapeutics and imaging applications. Int. J. Nanomed..

[38] Zaman M.S., Chauhan N., Yallapu M.M., Gara R.K., Maher D.M., Kumari S., Sikander M., Khan S., Zafar N., Jaggi M. (2016). Curcumin Nanoformulation for Cervical Cancer Treatment. Sci. Rep..

[39] Wang Y.J., Pan M.H., Cheng A.L., Lin L.I., Ho Y.S., Hsieh C.Y., Lin J.K. (1997). Stability of curcumin in buffer solutions and characterization of its degradation products. J. Pharm. Biomed. Anal..

[40] Anand P., Kunnumakkara A.B., Newman R.A., Aggarwal B.B. (2007). Bioavailability of curcumin: Problems and promises. Mol. Pharm..

[41] Greenwald R.B., Pendri A., Conover C.D., Lee C., Choe Y.H., Gilbert C., Martinez A., Xia J., Wu D., Hsue M. (1998). Camptothecin-20-PEG ester transport forms: The effect of spacer groups on antitumor activity. Bioorg. Med. Chem..

[42] Yurkovetskiy A.V., Hiller A., Syed S., Yin M., Lu X.M., Fischman A.J., Papisov M.I. (2004). Synthesis of a macromolecular camptothecin conjugate with dual phase drug release. Mol. Pharm..

[43] Dey S., Sreenivasan K. (2015). Conjugating curcumin to water soluble polymer stabilized gold nanoparticles via pH responsive succinate linker. J. Mater. Chem. B.

[44] Kim C.Y., Bordenave N., Ferruzzi M.G., Safavy A., Kim K.H. (2011). Modification of curcumin with polyethylene glycol enhances the delivery of curcumin in preadipocytes and its antiadipogenic property. J. Agric. Food Chem..

[45] Zhang G., Li X., Liao Q., Liu Y., Xi K., Huang W., Jia X. (2018). Water-dispersible PEG-curcumin/amine-functionalized covalent organic framework nanocomposites as smart carriers for in vivo drug delivery. Nat. Commun..

[46] Somani S., Laskar P., Altwaijry N., Kewcharoenvong P., Irving C., Robb G., Pickard B.S., Dufes C. (2018). PEGylation of polypropylenimine dendrimers: Effects on cytotoxicity, DNA condensation, gene delivery and expression in cancer cells. Sci. Rep..

[47] Laskar P., Saha B., Ghosh S.K., Dey J. (2015). PEG based random copolymer micelles as drug carriers: The effect of hydrophobe content on drug solubilization and cytotoxicity. Rsc Adv..

[48] Meewan J., Somani S., Laskar P., Irving C., Mullin M., Woods S., Roberts C.W., Alzahrani A.R., Ferro V.A., McGill S. (2022). Limited impact of the protein Corona on the cellular uptake of PEGylated zein micelles by melanoma cancer cells. Pharmaceutics.

[49] Chowdhury P., Nagesh P.K.B., Hatami E., Wagh S., Dan N., Tripathi M.K., Khan S., Hafeez B.B., Meibohm B., Chauhan S.C. (2019). Tannic acid-inspired paclitaxel nanoparticles for enhanced anticancer effects in breast cancer cells. J. Colloid Interface Sci..

[50] Nagesh P.K.B., Hatami E., Chowdhury P., Kashyap V.K., Khan S., Hafeez B.B., Chauhan S.C., Jaggi M., Yallapu M.M. (2018). Tannic Acid Induces Endoplasmic Reticulum Stress-Mediated Apoptosis in Prostate Cancer. Cancers.

[51] Lee H., Bang J.B., Na Y.G., Lee J.Y., Cho C.W., Baek J.S., Lee H.K. (2021). Development and Evaluation of Tannic Acid-Coated Nanosuspension for Enhancing Oral Bioavailability of Curcumin. Pharmaceutics.

[52] Nagesh P.K.B., Chowdhury P., Hatami E., Kumari S., Kashyap V.K., Tripathi M.K., Wagh S., Meibohm B., Chauhan S.C., Jaggi M. (2019). Cross-Linked Polyphenol-Based Drug Nano-Self-Assemblies Engineered to Blockade Prostate Cancer Senescence. ACS Appl. Mater. Interfaces.

[53] Al Nakeeb N., Nischang I., Schmidt B. (2019). Tannic Acid-Mediated Aggregate Stabilization of Poly(N-vinylpyrrolidone)-b-poly(oligo (ethylene glycol) methyl ether methacrylate) Double Hydrophilic Block Copolymers. Nanomaterials.

[54] Liu Z., Fan H., Li W., Bai G., Li X., Zhao N., Xu J., Zhou F., Guo X., Dai B. (2019). Competitive self-assembly driven as a route to control the morphology of poly(tannic acid) assemblies. Nanoscale.

[55] Smith R.A., Walker R.C., Levit S.L., Tang C. (2019). Single-Step Self-Assembly and Physical Crosslinking of PEGylated Chitosan Nanoparticles by Tannic Acid. Polymers.

[56] Liu X., O S., Yu B., Liu Y., Huang K., Gong J., Zheng S., Li Z., Li H., Jiang H. (2010). PharmMapper server: A web server for potential drug target identification using pharmacophore mapping approach. Nucleic Acids Res..

[57] Dhasmana A., Uniyal S., Anukriti, Kashyap V.K., Somvanshi P., Gupta M., Bhardwaj U., Jaggi M., Yallapu M.M., Haque S. (2020). Topological and system-level protein interaction network (PIN) analyses to deduce molecular mechanism of curcumin. Sci. Rep..

[58] Dhasmana A., Uniyal S., Somvanshi P., Bhardwaj U., Gupta M., Haque S., Lohani M., Kumar D., Ruokolainen J., Kesari K.K. (2019). Investigation of Precise Molecular Mechanistic Action of Tobacco-Associated Carcinogen ‘NNK’ Induced Carcinogenesis: A System Biology Approach. Genes.

[59] Chen Y.N., Peng L., Liu T., Wang Y., Shi S., Wang H. (2016). Poly(vinyl alcohol)-Tannic Acid Hydrogels with Excellent Mechanical Properties and Shape Memory Behaviors. Acs Appl. Mater. Interfaces.

[60] Souza T.G., Ciminelli V.S., Mohallem N.D.S. (2016). A comparison of TEM and DLS methods to characterize size distribution of ceramic nanoparticles. Journal of Physics: Conference Series.

[61] Sikander M., Malik S., Khan S., Kumari S., Chauhan N., Khan P., Halaweish F.T., Chauhan B., Yallapu M.M., Jaggi M. (2019). Novel mechanistic insight into the anticancer activity of cucurbitacin D against pancreatic cancer (Cuc D attenuates pancreatic cancer). Cells.

[62] Gupta B.K., Maher D.M., Ebeling M.C., Stephenson P.D., Puumala S.E., Koch M.R., Aburatani H., Jaggi M., Chauhan S.C. (2014). Functions and regulation of MUC13 mucin in colon cancer cells. J. Gastroenterol..

[63] Sapra P., Zhao H., Mehlig M., Malaby J., Kraft P., Longley C., Greenberger L.M., Horak I.D. (2008). Novel delivery of SN38 markedly inhibits tumor growth in xenografts, including a camptothecin-11-refractory model. Clin. Cancer Res..

[64] Friedman L., Lin L., Ball S., Bekaii-Saab T., Fuchs J., Li P.K., Li C., Lin J. (2009). Curcumin analogues exhibit enhanced growth suppressive activity in human pancreatic cancer cells. Anti-Cancer Drugs.

[65] Li X.Q., Wen H.Y., Dong H.Q., Xue W.M., Pauletti G.M., Cai X.J., Xia W.J., Shi D., Li Y.Y. (2011). Self-assembling nanomicelles of a novel camptothecin prodrug engineered with a redox-responsive release mechanism. Chem. Commun..

[66] Ma W., Cheetham A.G., Cui H. (2016). Building Nanostructures with Drugs. Nano Today.

[67] Zhou Y., Fan S., Feng L., Huang X., Chen X. (2021). Manipulating intratumoral fenton chemistry for enhanced chemodynamic and chemodynamic-synergized multimodal therapy. Adv. Mater..

[68] Feng L., Zhao R., Yang L., Liu B., Dong S., Qian C., Liu J., Zhao Y. (2023). Tumor-Specific NIR-Activatable Nanoreactor for Self-Enhanced Multimodal Imaging and Cancer Phototherapy. ACS Nano.

[69] Yang L., Shi R., Zhao R., Zhu Y., Liu B., Gai S., Feng L. (2022). Near-infrared upconversion mesoporous tin dioxide theranostic nanocapsules for synergetic cancer chemophototherapy. ACS Appl. Mater. Interfaces.

