# Supplementary material for: Glutathione-Responsive Tannic Acid-Assisted FRET Nanomedicine for Cancer Therapy
Source: Pharmaceutics. 2023 Apr 24;15(5):1326. doi: 10.3390/pharmaceutics15051326 (PMC10222396; doi:10.3390/pharmaceutics15051326)
Supplement: Supplementary file 1 [file pharmaceutics-15-01326-s001.zip › pharmaceutics-2259280-supplementary.pdf]

pharmaceutics

Supplementary Information

Glutathione-responsive tannic acid-assisted FRET nanomedicine for cancer therapy

Partha Laskar[1,2,3], Anupam Dhasmana[1,2,4], Sudhir Kotnala[1,2], Meena Jaggi[1,2], Murali M. Yallapu[1,2*] and Subhash C. Chauhan[1,2*]

[1]Department of Immunology and Microbiology, School of Medicine, University of Texas Rio Grande Valley, McAllen, TX 78504, USA

[2]South Texas Center of Excellence in Cancer Research, School of Medicine, University of Texas Rio Grande Valley, McAllen, TX 78504, USA

[3]Macromolecular Chemistry, Department of Chemistry, The Ångström Laboratory, Uppsala University, Sweden

[4]Himalayan School of Biosciences and Cancer Research Institute, Swami Rama Himalayan University, Dehradun, India

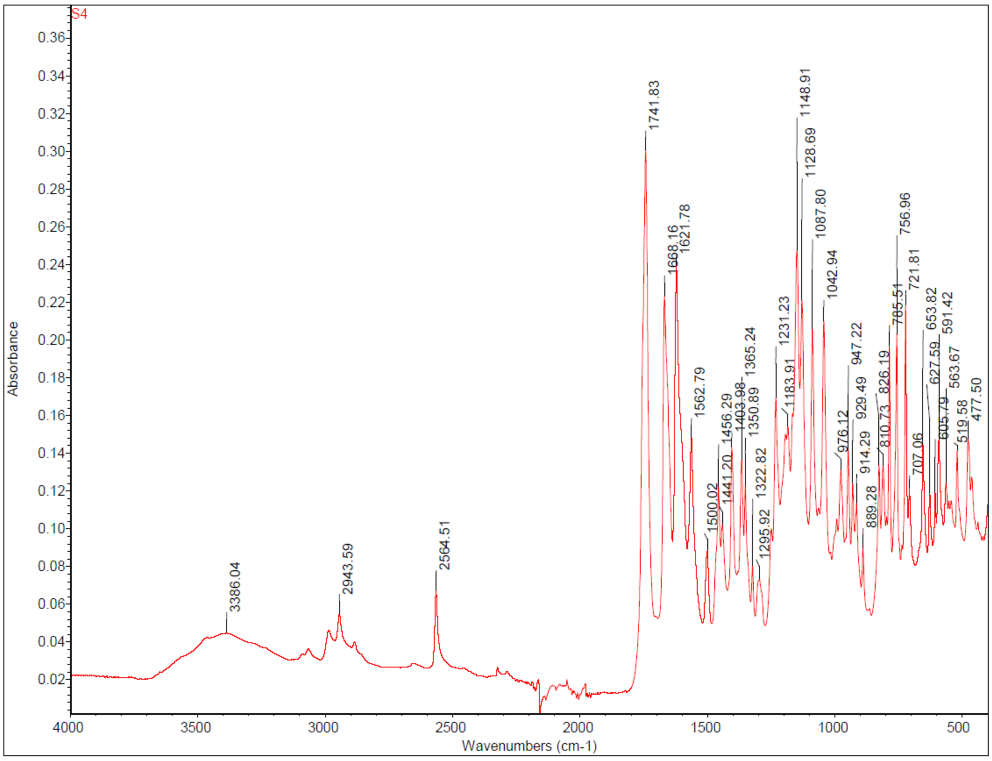

Figure S1. FTIR spectra of CPTSH.

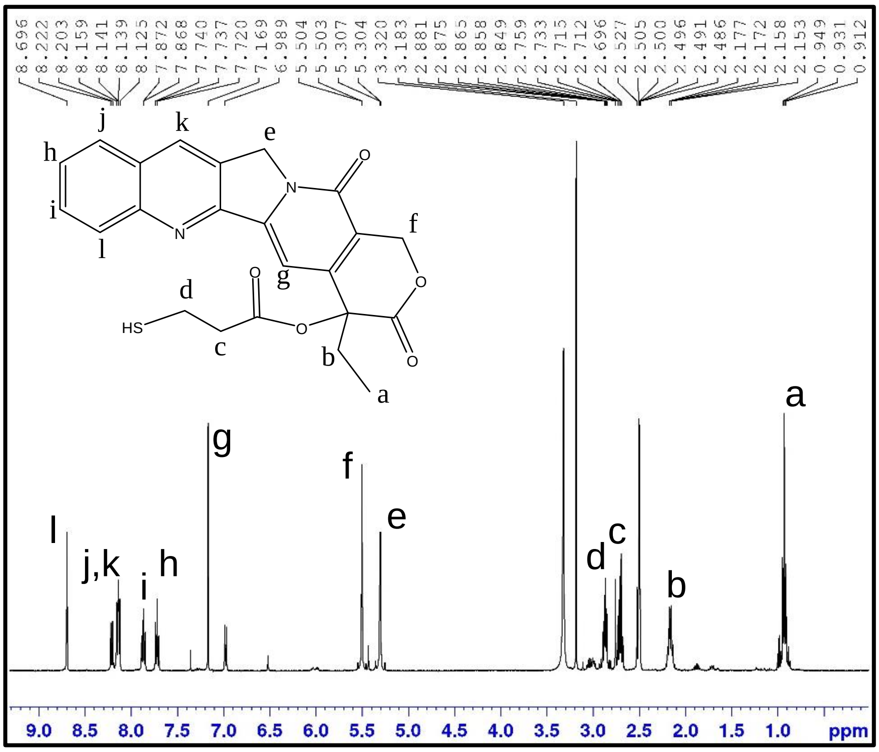

Figure S2. ^{1}H-NMR spectrum of CPTSH (in DMSO-d6, 400 MHz).

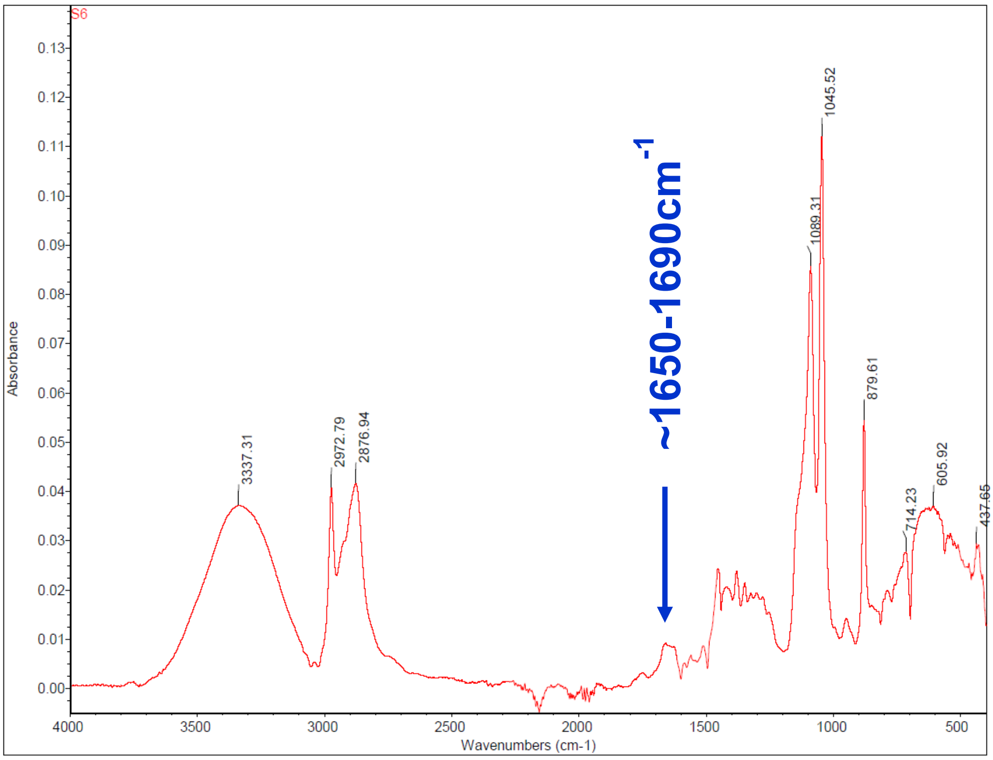

Figure S3. FTIR of dual drug (CPT and CUR) conjugated PEG-based polymer, CPT-S-S-PEG-CUR.

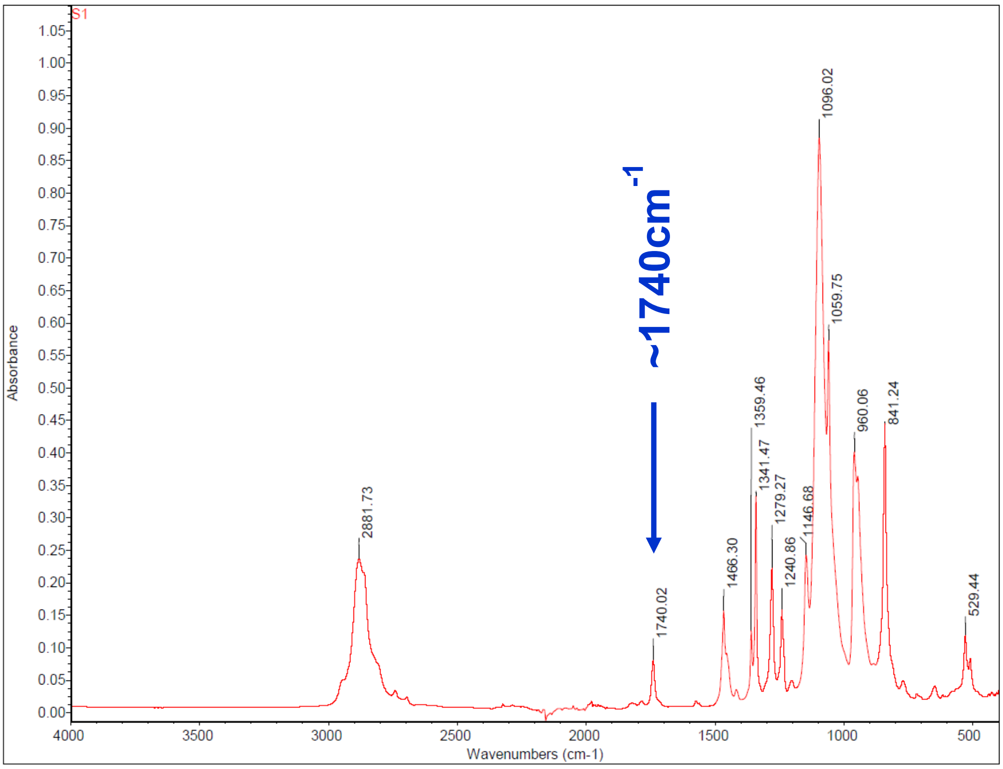

Figure S4. FTIR spectra of starting material OPSS-PEG-SCM.

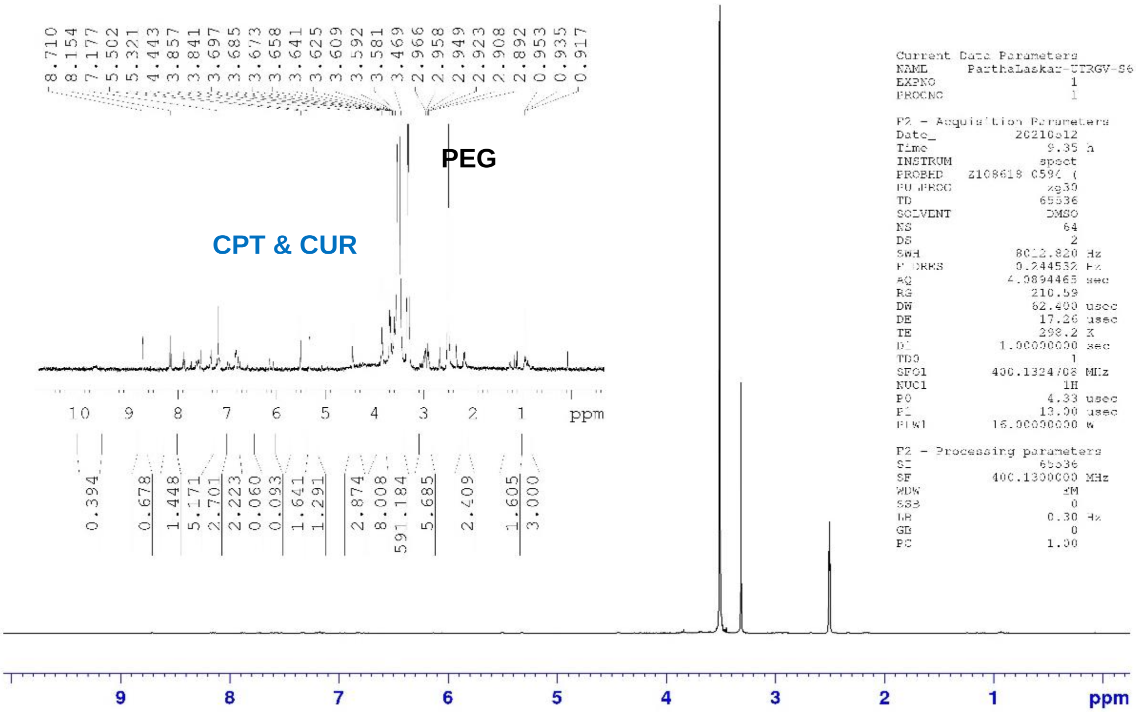

Figure S5. ^{1}H-NMR spectrum of dual drug (CPT and CUR) conjugated PEG-based polymer CPT-S-S-PEG-CUR (in DMSO-d6, 400 MHz).

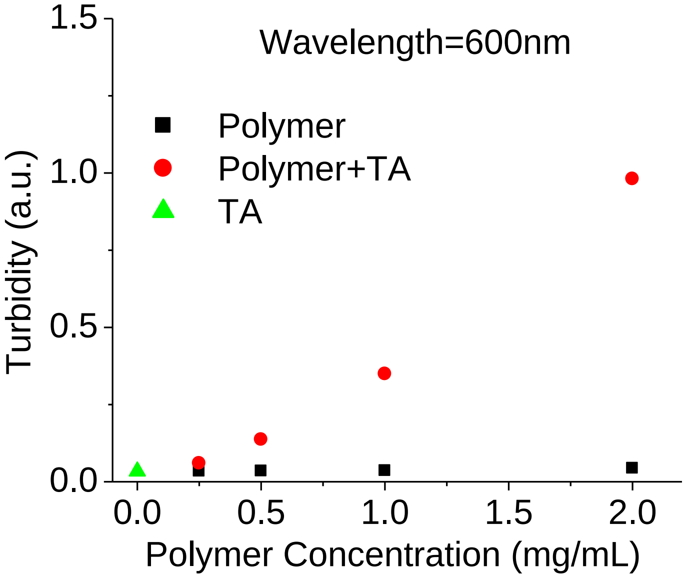

Figure S6. Turbidity (wavelength=600 nm) of only TA solution (5 mg/mL) and CPT-S-S-PEG-CUR solution (0.25, 0.5, 1.0, and 2.0 mg/mL) in water in absence and presence of TA (5 mg/mL).

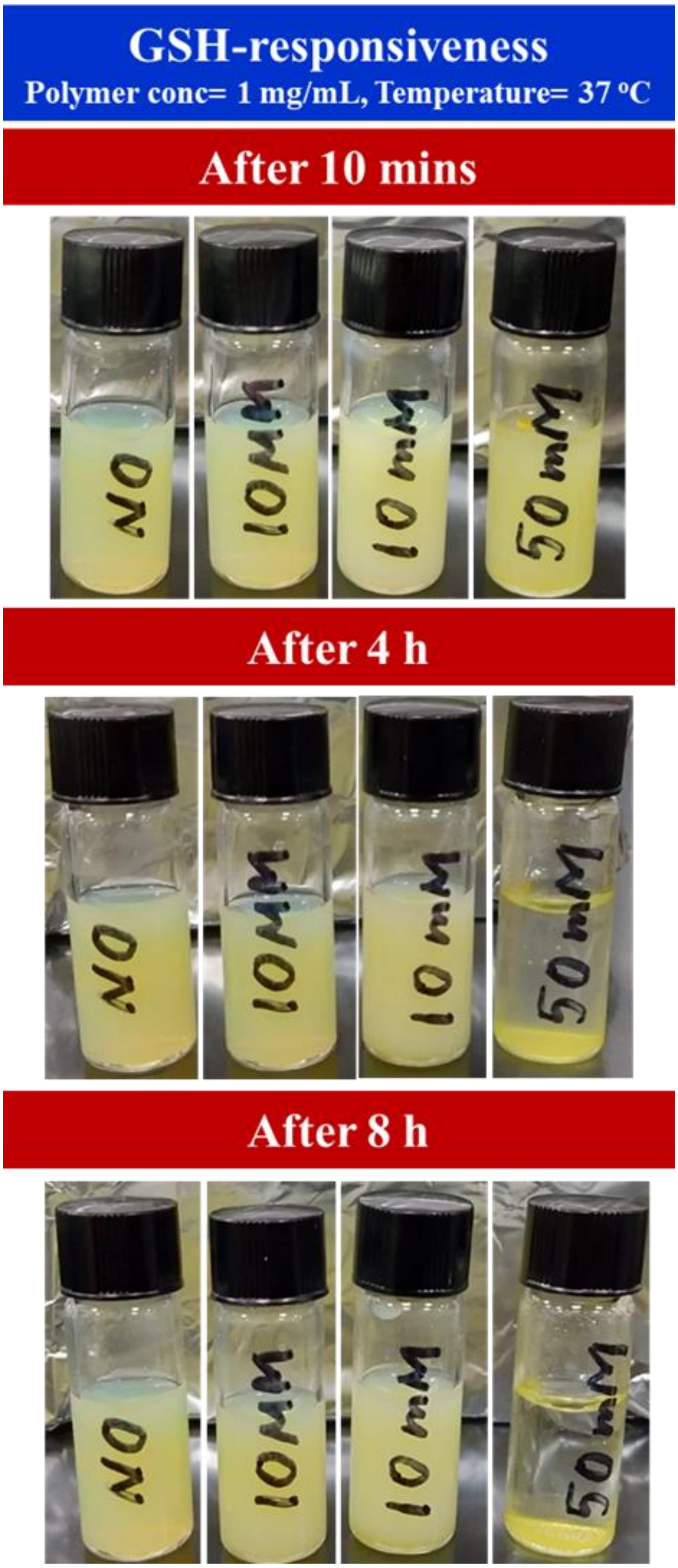

Figure S7. Photographs of polymer (1 mg/mL)-TA nanoassemblies in absence and presence of various GSH concentration (10 μM, 10 mM, and 50 mM) in water at 37 °C.

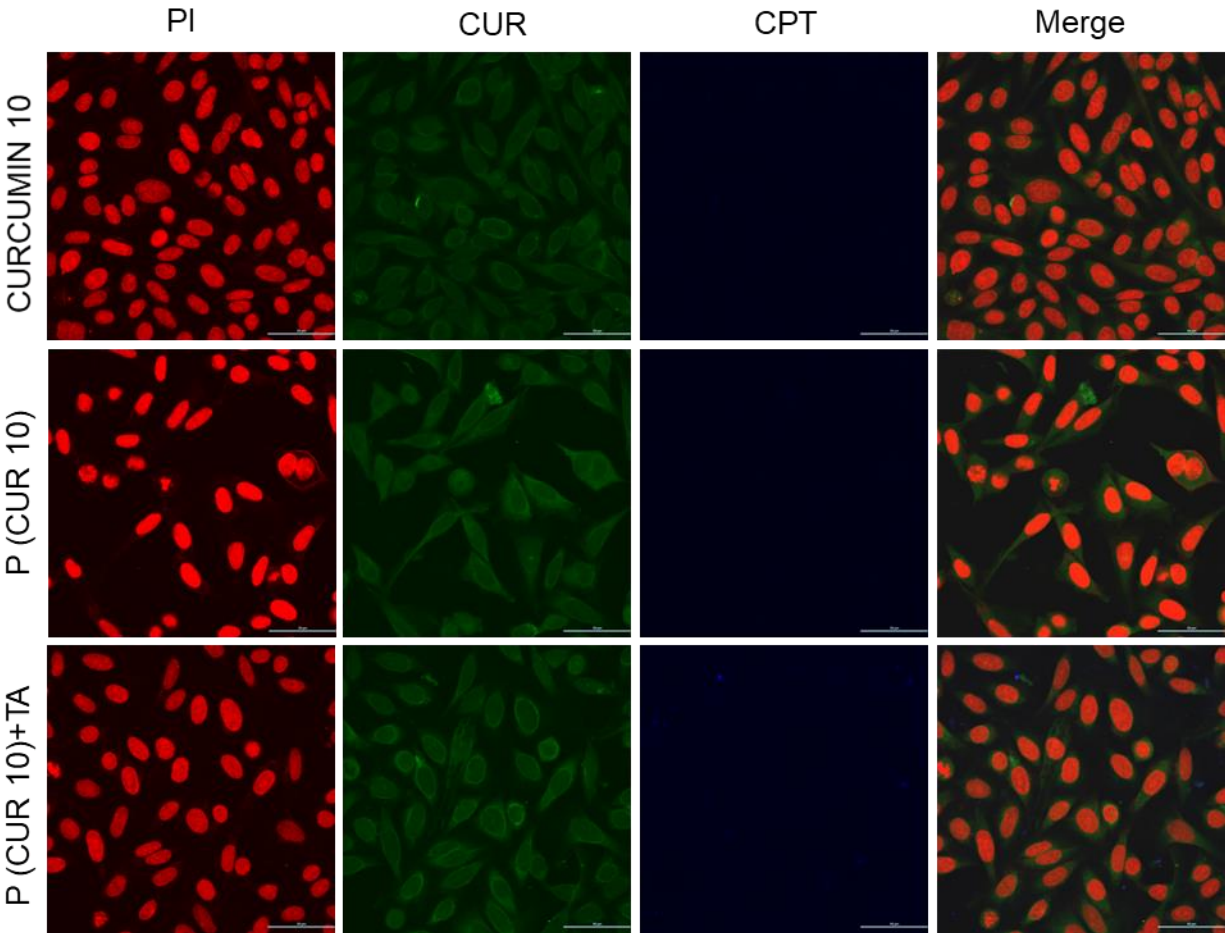

Figure S8. Cellular uptake of only drug CUR, and polymer CUR-PEG-S-S-CPT in absence and presence of TA by AsPC1 pancreatic cancer at 10μM CUR-equivalent concentrations after 4h of incubation (magnification: ×40, Zoom: 2.08, scale bar: 50 μm).

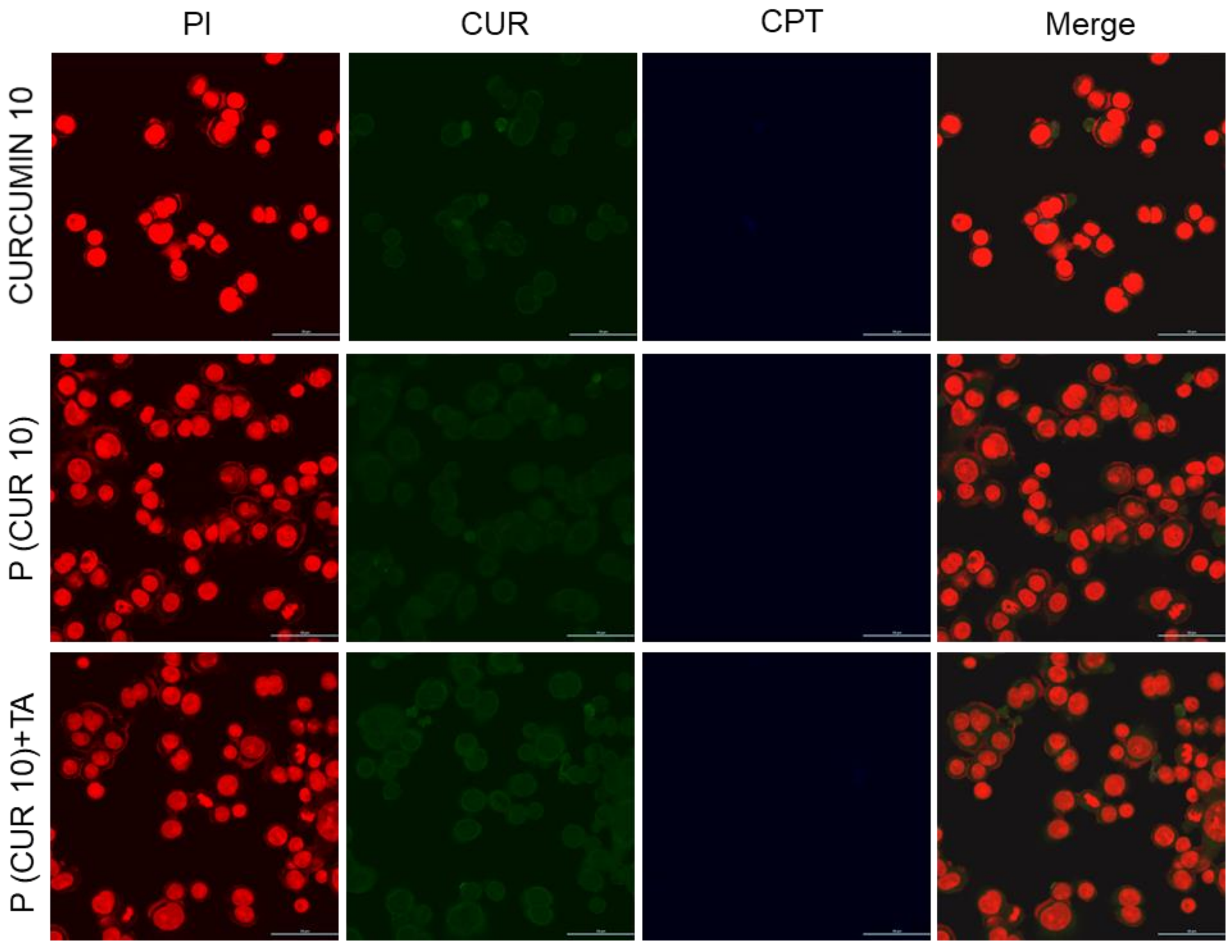

Figure S9. Cellular uptake of only drug CUR, and polymer CUR-PEG-S-S-CPT in absence and presence of TA by SW480 colon cancer cell at 10μM CUR-equivalent concentrations after 4h of incubation (magnification: ×40, Zoom: 2.08, scale bar: 50 μm).

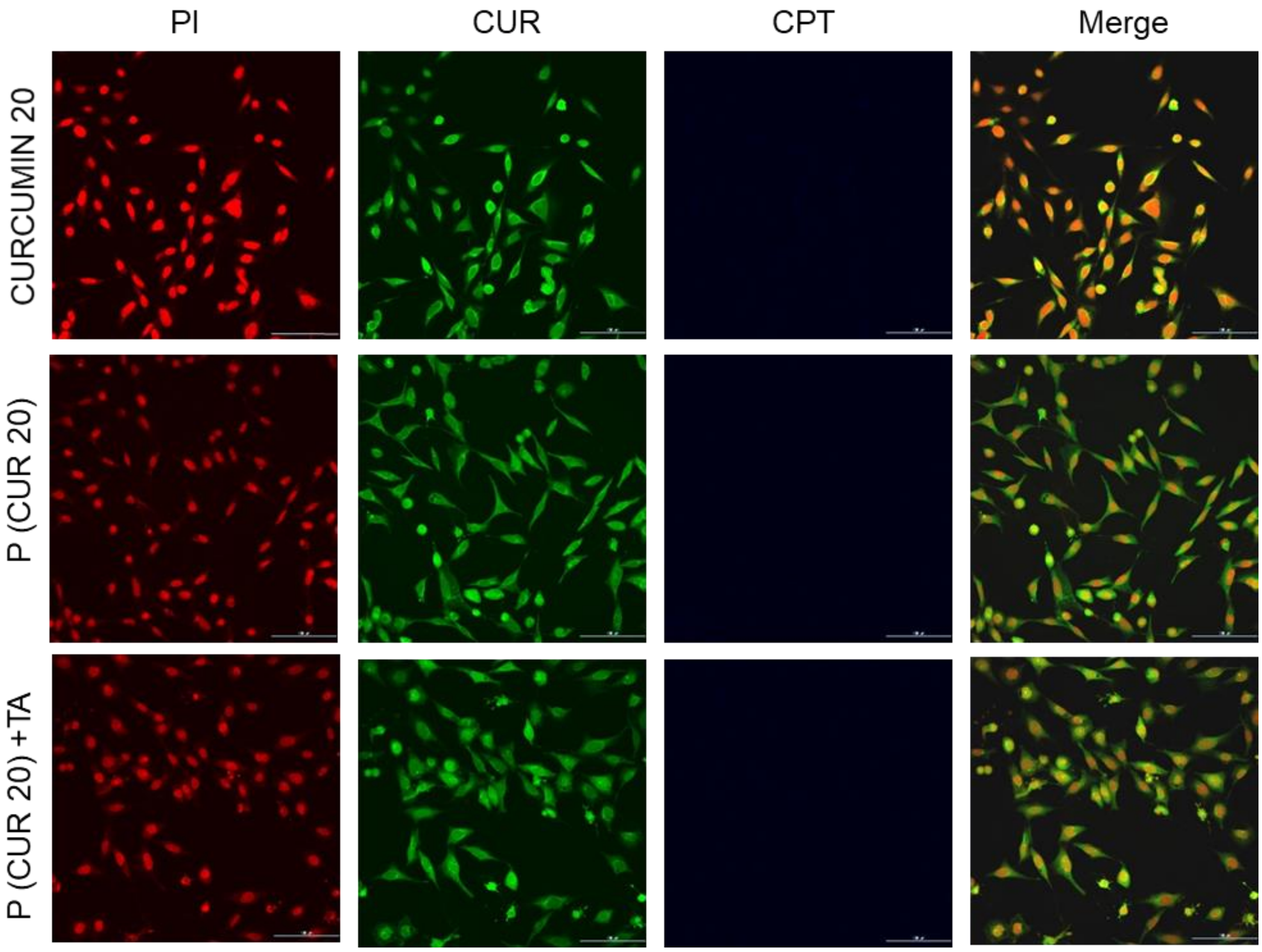

Figure S10. Cellular uptake of only drug CUR, and polymer CUR-PEG-S-S-CPT in absence and presence of TA by AsPC1 pancreatic cancer at 20μM CUR-equivalent concentrations after 12h of incubation (magnification: ×40, scale bar: 100 μm).

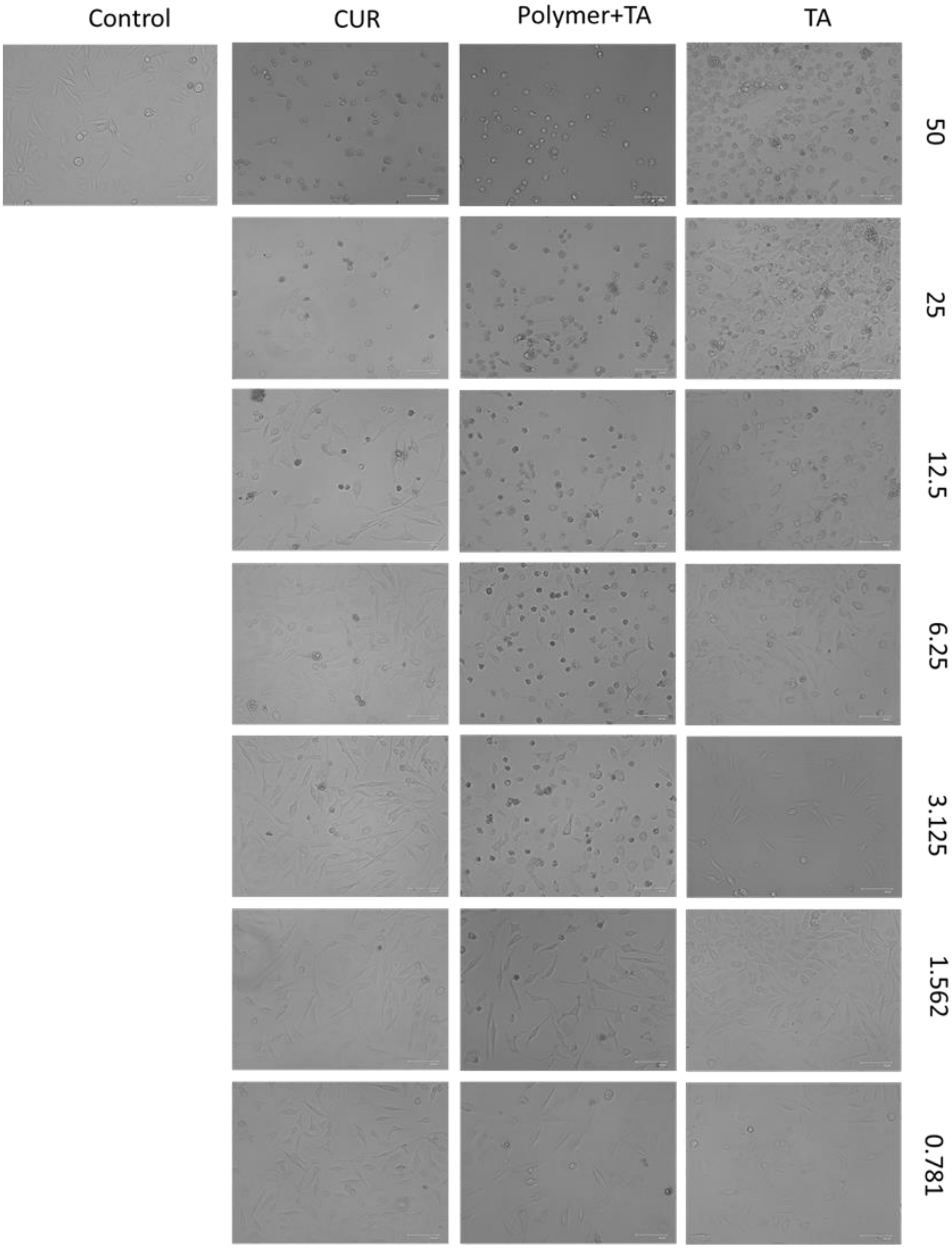

Figure S11. Images of the AsPC1 cells treated with the CUR, Polymer (CUR equivalent) +TA, and TA (Polymer+TA equivalent) after 48 h.

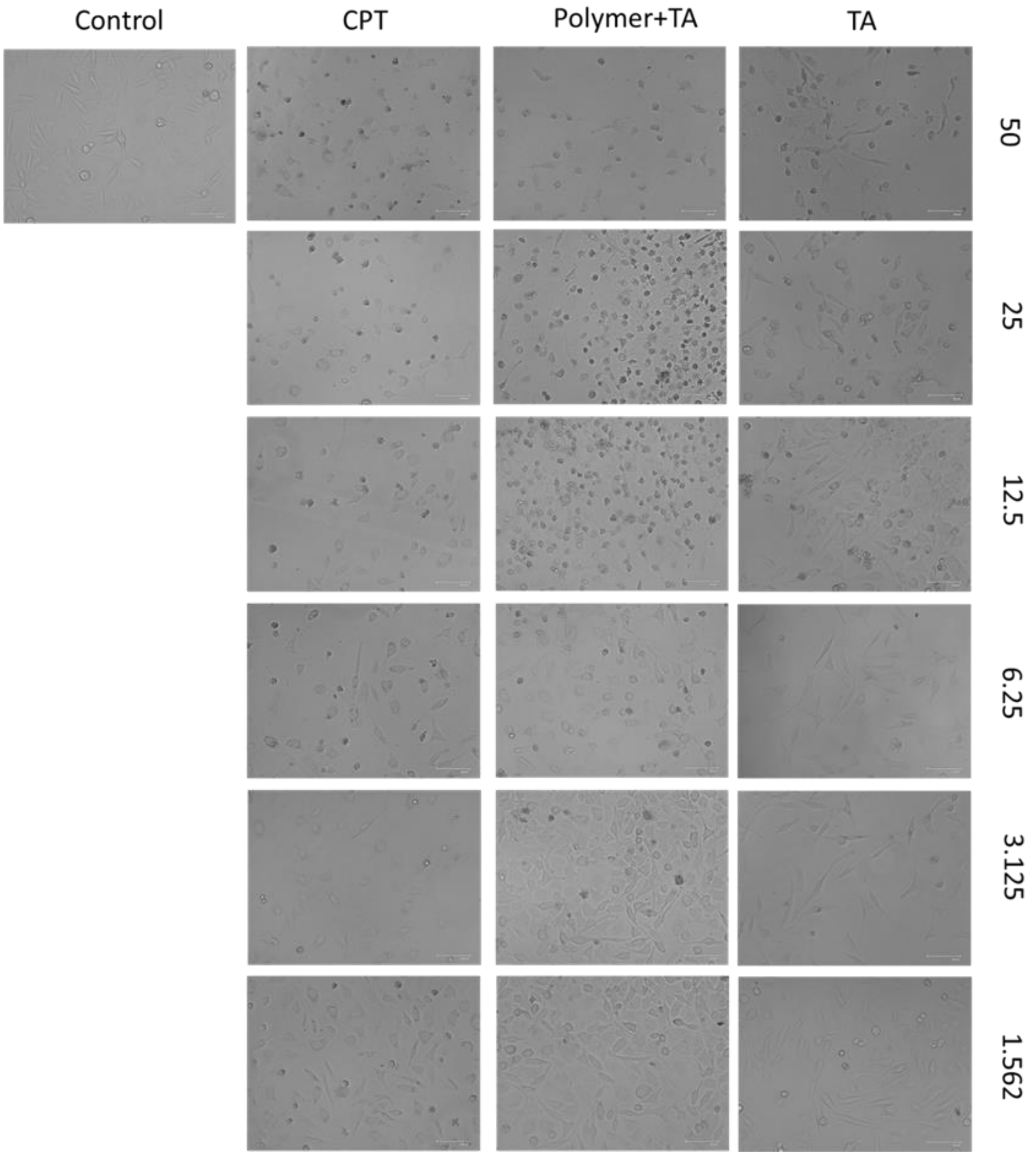

Figure S12. Images of the AsPC1 cells treated with the CPT, Polymer (CPT equivalent) +TA, and TA (Polymer+TA equivalent) after 48 h.

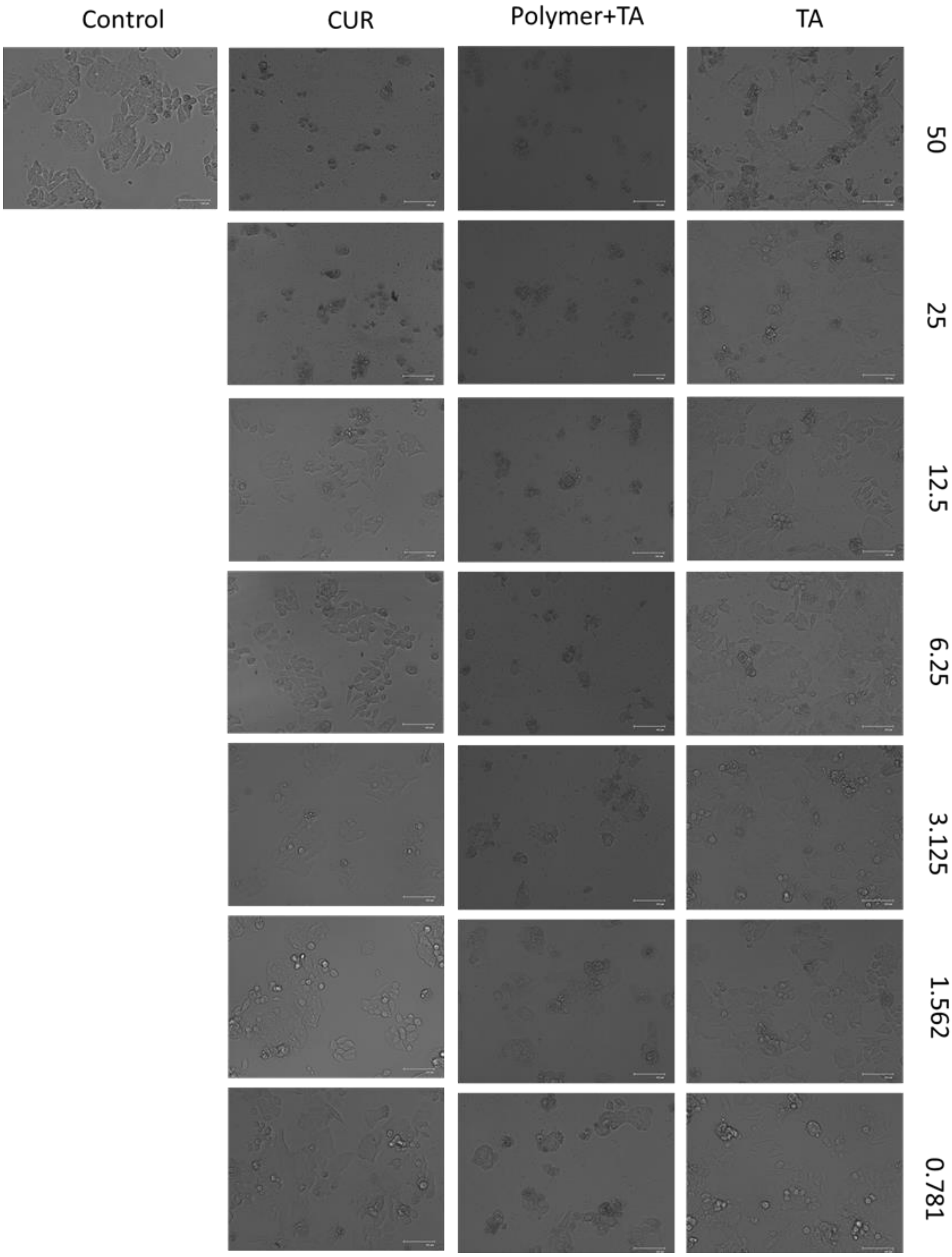

Figure S13. Images of the SW480 cells treated with the CUR, Polymer (CUR equivalent) +TA, and TA (Polymer+TA equivalent) after 48 h.

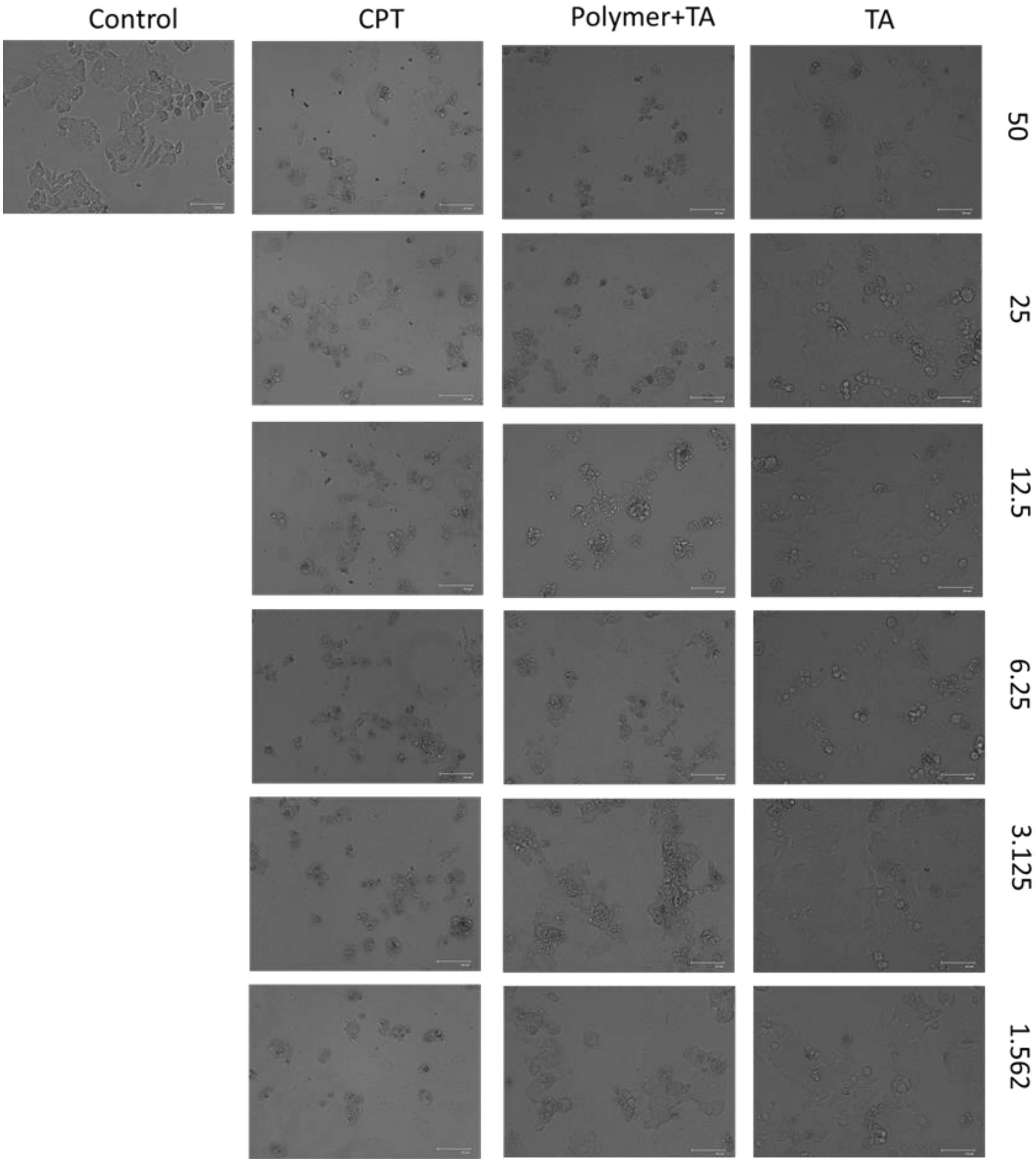

Figure S14. Images of the SW480 cells treated with the CPT, Polymer (CPT equivalent) +TA, and TA (Polymer+TA equivalent) after 48 h.

A

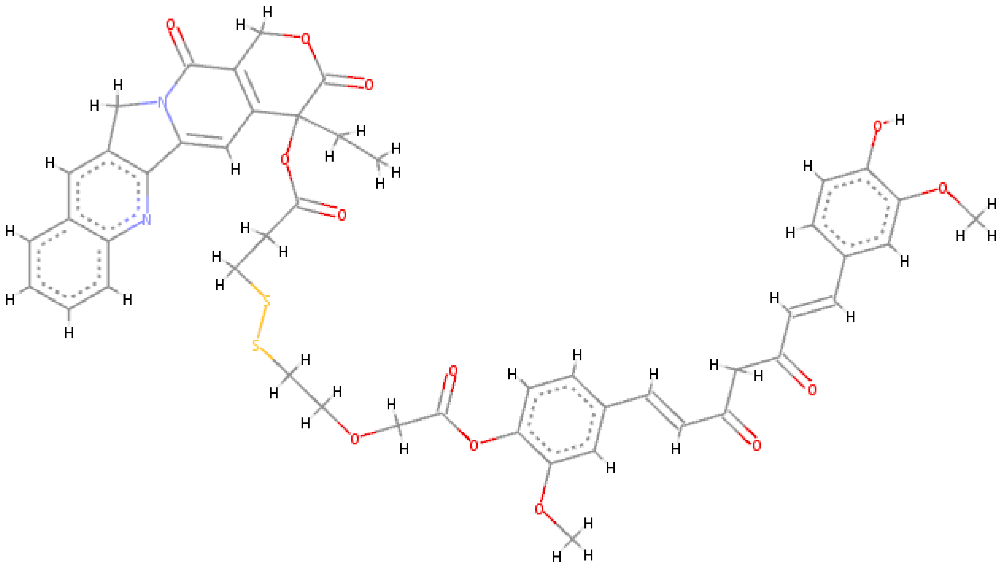

B **Physical Properties of Test Compound:**

Molecular formula: $C_{48}H_{44}N_2O_{13}S_2$

Molecular Weight: 920.99856

ALogP: 6.564

Rotatable Bonds: 22

Acceptors: 16

Donors: 1

Figure S15. 2D chemical representative diagram (A) and physical properties (B) of test compound.
